# An English list of trait words including valence, social desirability, and observability ratings

**DOI:** 10.3758/s13428-022-01921-5

**Published:** 2022-08-12

**Authors:** Sara Britz, Lena Rader, Siegfried Gauggel, Verena Mainz

**Affiliations:** Institute of Medical Psychology and Medical Sociology, University Hospital of the RWTH Aachen University, Aachen, Germany

**Keywords:** Database, Trait adjectives, Valence, Social desirability, Observability

## Abstract

To enable flexible and controlled research on personality, information processing, and interactions in socio-emotional contexts, the availability of highly controlled stimulus material, especially trait words and related attributes, is indispensable. Existing word databases contain mainly nouns and rating dimensions, and their role in studies within socio-emotional contexts are limited. This study aimed to create an English list of traits (ELoT), a database containing 500 trait adjectives rated by a large sample (*n* = 822, 57.42% female). The rating categories refer to the perceived valence associated with the traits and their social desirability and observability. Participants of different ages (18 to 65 years of age) and educational levels rated the words in an online survey. Both valence and social desirability ratings showed a bimodal distribution, indicating that most traits were rated either positive (respectively socially desirable) or negative (respectively socially undesirable), with fewer words rated as neutral. For observability, a bell-shaped distribution was found. Results indicated a strong association between valence and social desirability, whereas observability ratings were only moderately associated with the other ratings. Valence and social desirability ratings were not related to participants’ age or gender, but observability ratings were different for females and males, and for younger, middle-aged, and older participants. The ELoT is an extensive, freely available database of trait norms. The large sample and the balanced age and gender distributions allow to account for age- and gender-specific effects during stimulus selection.

**Supplementary Information:**

The online version contains supplementary material available at 10.3758/s13428-022-01921-5.

## Introduction

In experimental studies, the use of verbal material is frequently used to answer a wide variety of research questions. In recent years, some databases and norms on verbal material have been made available in different languages and with a focus on different parts of speech, such as nouns, verbs, or adjectives (e.g., Bradley & Lang, 1999; Britz et al., 2019; Chandler, 2018; Gilet et al., 2012; Grühn, 2016; Lahl et al., 2009; Mohammad, 2018; Montefinese et al., 2014; Verheyen et al., 2020). These databases allow researchers to control objective word characteristics that can influence information processing (such as word length and word frequency) and also to intentionally use specific attributes of the words (such as imageability and arousal) in experimental manipulations in order to influence cognitive processing (e.g., Garcia et al., 2012; Kauschke et al., 2019; Rubin & Friendly, 1986).

Although there is consensus that certain stimulus attributes can and should be controlled (Kanske & Kotz, 2010), only nouns and psycholinguistic attributes are primarily being studied. In the currently available databases on word norms, efforts have been made to integrate valence (e.g., Warriner et al., 2013), imageability (e.g., Grühn, 2016), arousal (e.g., Schmidtke et al., 2014), concreteness (e.g., Grühn, 2016), and dominance (e.g., Mohammad, 2018) in addition to psycholinguistic variables. Still most progress in the inclusion of word characteristics is evident regarding the emotional valence of words and/or their arousal value (e.g., Grühn, 2016). Nevertheless, especially in research investigating emotions or emotionally affected humans, such as patients with mental disorders or humans in social interactions, additional stimulus attributes are needed but are rarely included. In the long tradition of personality research, appropriately validated instruments for measuring personality dimensions have been developed through psycholinguistic and lexical approaches (e.g., Costa & McCrae, 1992).

It is surprising that so far in this research the use of adjectives that depict personality dimensions has received little attention in experimental studies that use lexical databases. In fact, there are few lexical databases of adjectives that include additional information on valence and other socio-emotional attributes that could be used in experimental research for manipulation and stimulus control (Grühn, 2016; Montefinese et al., 2014). This can possibly be explained by the fact that the majority of the research that is more concerned with the exploration of stimulus material originates from experimental cognition, emotion research and the associated information processing, and less from research on the influence of personality on pathologies in the socio-emotional context. The aim of the present study was to create an English-language database of trait adjectives which would include, in addition to psycholinguistic characteristics, perceived valence and social desirability as well as the degree to which the traits can be seen by an outside observer.

### Database of adjectives

This study aimed to develop a database of adjectives because, as noted above, most existing databases contain only nouns, and this restricts the selection of stimuli in certain research areas. Personality traits can be defined as “the relatively enduring patterns of thoughts, feelings, and behaviors that reflect the tendency to respond in certain ways under certain circumstances” (Roberts et al., 2009). Adjectives, moreover, have been described as perfectly suited for investigating the interface between cognition and emotion (Verheyen et al., 2020). It would seem worthwhile, therefore, to enrich databases by including adjectives. This would facilitate more flexible and controlled research on both personality-related issues and self-referential information processing and its interactions in socio-emotional contexts. The database that was constructed builds on previous research in which the authors created a German-language database (Britz et al., 2019), which included traits for experimental investigations of personality related to the Big Five framework (Costa & McCrae, 1992), self-referential processes (Forster et al., 2019; Mainz et al., 2020; Gosling et al., 1998), and other socio-emotional research (Wentura et al., 2000). The research was built around the following foci:

### Psycholinguistic attributes

The trait attributes that the study focused on are closely linked to the stimulus attributes used in psycholinguistic research (e.g., frequency of occurrence, word length). They can be used in experimental studies to control for their influence on cognitive processing (i.e., word features that influence attention and memory performance, e.g., Gorman, 1961). For example, it has been shown that differences in the valence of such words might correspond to differences in the frequencies with which these words occur in the language (e.g., Bliss et al., 2012). Similarly, positively and negatively valenced words seem to differ systematically in word length (Grühn, 2016). It is, therefore, important to provide the most important psycholinguistic characteristics of the words in a database that also includes ratings on valence, social desirability, and observability.

### Valence

The valence of words was of focus in the study because previous research has demonstrated the importance of emotionally and socially relevant characteristics of words (e.g., Grühn & Sharifian, 2016). Some of the early databases that assessed the likability of person-descriptive words (e.g., Anderson, 1968) found that likability ratings were bimodal, containing mostly negative but also some positive and a few neutral words (see Chandler, 2018). Comprehensive databases that have included norms on valence include the *Dictionary of Affect in Language* (Whissell, 1989); the revised edition of the *Dictionary of Affect in Language* (Whissell, 2009); a database that Warriner et al. (2013) created, which includes valence, arousal, and dominance ratings; Grühn’s (2016) *EMOTE*, which includes valence, arousal, and emotionality ratings among others; Mohammad’s (2018) *NRC VAD Lexicon* with valence, arousal, and dominance ratings; and Bradley and Lang’s (1999) *Affective Norms for English Words* (ANEW) with valence and arousal ratings. Efforts have been made to validate the ANEW in different languages, such as European Portuguese (Soares et al., 2012) and Italian (Montefinese et al., 2014). The latter included larger samples, which allowed researchers to investigate effects of person characteristics (e.g., gender) on valence ratings. Some databases have also examined the influence of age on valence ratings (Gilet et al., 2012; Grühn & Smith, 2008; Söderholm et al., 2013). Nevertheless, either the majority of words included in the databases that included valence attributes were nouns rather than adjectives, or the database was not assembled in an English-speaking context, which is a gap in the literature that the current study aimed to fill.

### Social desirability

The need to include social desirability ratings in a trait-stimuli database originated in the authors’ experimental research on the processing of self-referential information (Forster et al., 2019). The possibility that participants might respond in a socially desirable manner when ascribing positively and negatively valenced traits to themselves motivated the authors to create a German database for trait adjectives containing both valence and social desirability ratings (Britz et al., 2019). Studies have shown that people often exhibit socially desirable response behavior in order to make a good impression that comes at the expense of honest and accurate responses, and this tendency biases self-report measures of personality traits (Holtgraves, 2004; Rosen, 1956). Therefore, and a common research practice up to this point, research attempts to determine the extent to which a tendency to respond in a socially desirable way may have influenced participants’ responses on questionnaire studies in which covariate scores were used in the data analyses (e.g., Crowne & Marlowe, 1960; Jacobson et al., 1977; Konstabel et al., 2006; Stöber, 2001). For that reason, and similar to Bochner and Van Zyl’s (1985) use of social desirability ratings and Anderson’s (1968) use of likableness ratings, a database containing valence and social desirability ratings was established in a German sample in research on improved item selection (Britz et al., 2019). Although valence and social desirability are strongly linked, the two kinds of ratings (i.e., positively rated trait adjectives are for the most regarded as socially desirable and negatively rated traits as socially undesirable) cannot necessarily be regarded as measuring the same theoretical construct; in fact, for some words, weak or no correlation between the two kinds of ratings have been found (Britz et al., 2019). The German database allows more control over the stimuli in experiments in which personality traits are judged by respondents in different kinds of research designs (e.g., round robin designs, designs involving perspective changes during social interaction). In the present study, the trait ratings were adapted for native English speakers and again the focus was on ratings of social desirability that consider a social reference norm, i.e., what the person thinks that society considers desirable, which may however diverge from what raters personally would consider as favorable or unfavorable (Hager & Hasselhorn, 1994). See Britz et al. (2019) for a more detailed description of the relevance of social desirability and social reference norms.

### Observability

Finally, this study also includes observability as a rating dimension in the database. Observability, also termed *visibility* (Watson et al., 2000), is the extent to which a personality trait comprises a clear and frequent behavioral manifestation (Watson et al., 2000). Hence, an observable trait refers to one that is readily observed (e.g., talkative), whereas difficult-to-observe traits are more internal, subjective, and difficult to perceive (e.g., intelligent). In personality and socio-emotional research, the observability of traits and behaviors has previously been investigated (e.g., Vazire, 2010). It has been shown that the more visible a trait is, the more accurately it can be judged in interjudge agreements, which is called the *trait-visibility effect*. Depending on the focus of a particular study, it would seem reasonable to include those stimuli that can better be assessed from the perspective of the self or others, as in knowledge asymmetry between traits that are high or low in observability (Vazire, 2010). Further, it has been shown that it is easier to capture observable characteristics from the external perspective, and this leads to more accurate judgments, whereas less visible characteristics are judged more accurately from the point of view of the self probably not only because they are more salient but also because such traits are more significant for oneself (Vazire, 2010). It has, for instance, been shown that self-other agreements are influenced by a trait’s observability and affectivity (Watson et al., 2000) and by its social desirability (John & Robins, 1993). The highest agreement between self- and other-judgements has been shown for traits that are high in observability and low in affectivity (Watson et al., 2000) and social desirability (John & Robins, 1993). Importantly, however, trait observability and social desirability are regarded as separate concepts (Gosling et al., 1998). In sum, the integration of ratings of valence, social desirability, and observability improves stimulus selection in experimental studies using verbal material.

As a final point, the current study addressed some of the general limitations of databases that Bochner and Van Zyl (1985), Grühn and Smith (2008), and Fairfield et al. (2017) have discussed. For instance, researchers have relied on ratings of student samples or of well-educated respondents, and cross-cultural differences between word meanings and ratings in German and English remain so far unanswered in the current authors’ research (Britz et al., 2019).

Achieving a balance between sample size and a study’s practicality for the participants is an important consideration. In order to ensure an adequate study duration with the given number of ratings, the stimulus material is often divided among the study participants, so that each word is rated on all dimensions by only a portion of the participants. Additionally, researchers may want to address gender and age differences in the stimulus ratings, and this would require large samples in order to prevent power restrictions and to be able to make valid conclusions about individual words. With these considerations in mind, this study aimed to have a large sample of stimuli and three different rating dimensions.

## Methods

### Rating material

**Fig. 1:**
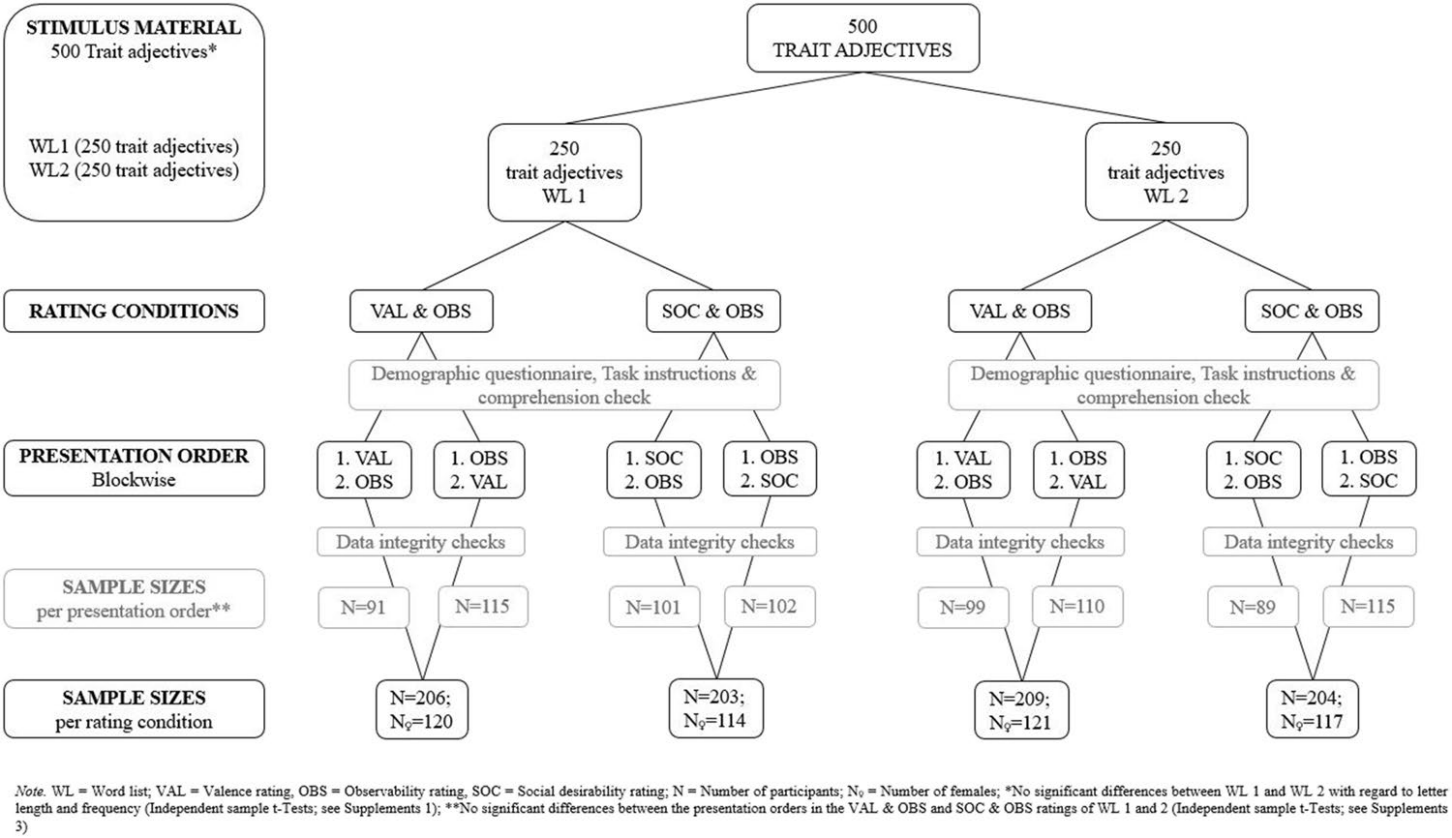
Study design

The words used in this study were taken from the Aachen List of Trait words (Britz et al., 2019), which contains 606 German adjectives. The words were translated from German into English using the translator Apps Leo (www.dict.leo.org) and PONS (http://pons.com). In case the same English translation was suggested for different German words, the word list was reduced. The word list was extended to include adjectives describing personality traits (e.g., artistic, careful, intelligent, social). The inclusion of these traits arose from studies investigating the clustering and factorial structure of traits that had been used in personality inventories (e.g., Big Five, NEO; Ashton et al., 2004; Wood et al., 2010). The final word list comprised 500 trait adjectives, each of which was assigned to one of two separate word lists through pseudo-randomization based on word length and alphabetical distribution (see Fig. 1).

Word frequency coefficients were derived from the Celex (Baayen et al., 1993) and the SUBTLEX-US databases. Zipf frequencies (Log 10 frequencies per one billion words) were taken from the SUBTLEX-US database, and Log10 frequencies per one million words were taken from the Celex database (CobLog; logarithm to base 10). The Zipf frequencies were chosen as the preferred index because it is independent of corpus size (Van Heuven et al., 2014; see Supplements 1 for the comparison of Word List 1 and Word List 2 on word length and frequency). The complete ELoT can be obtained from https://osf.io/23hc8/?view_only=46298b4d67d54fe58d5491f1b938e31f. There, psycholinguistic and frequency coefficients can be found in Database 5.

In addition to the 500 trait adjectives, ten non-words (randomly assembled letters, e.g., “northeloomed”) were generated using a Nonsense Word Generator (https://soybomb.com) and added to both of the word lists (see Supplements 2).

### Procedure

#### Online tool

The study was conducted via the survey platform Qualtrics (Qualtrics Labs Inc., Provo, Utah; https://qualtrics.com/), through which participants were recruited from 1st May to 9th November 2020. Qualtrics enables sampling through ESOMAR (European Society for Opinion and Marketing Research), which is a national association for market research. It manages the sampling procedure and allows integrity checks and pre-processing of the anonymous data to check for potential violations of inclusion criteria or deviating responses.

#### Sampling procedures

The recruitment of participants included screening criteria, quotas, and quality checks. The platform presented the study as eligible for users between 18 and 65 years of age and living in the United States. Potential participants could not access the study if they were using a mobile phone because doing so would have affected the presentation of the rating scale. Participants were also required to give informed consent.

Qualtrics randomly assigned participants to one of four conditions (Fig. 1). Data collection ceased when 200 participants per condition had been recruited. Participants in each condition were to be equally distributed across different age groups (from 18 to 65 in steps of ten) and gender (male and female). In case the age or gender quota had already been met in one condition, participants were either randomly assigned to another condition, or they were excluded from the study if all quotas had already been met. When data collection was completed, Qualtrics anonymously exported the data using randomized user IDs. Participants were excluded if they had invalid data (see *Data Integrity Checks*) and were then re-recruited through the platform aiming for the same gender, age, and study condition. Finally, participants were compensated with incentives distributed by Qualtrics’ ESOMAR-verified panel partners (https://esomar.org). For each 15 min of participation, participants received 60 panel points (ca. $2.50), which could then be exchanged in a point-based reward system for prizes and competitions from the different panel partners.

#### Study procedures

Once they had been assigned to a condition, participants received written explanations of the study’s purpose and procedure, and they gave written informed consent. For an overview of the study design, see Fig. 1. To keep the completion time per person as short as possible, each respondent rated only one of the word lists (Word List 1 or Word List 2). All words in a list were randomly presented to the participants. In one condition, either (a) social desirability (SOC) and observability (OBS), or (b) valence (VAL) and OBS were rated. Hence, participants rated 250 words per block for either VAL or SOC (depending on the condition), and then they rated the same 250 words for OBS. To avoid order effects, the order of the rating with which a condition started was randomized (see Supplements 3 for the analyses of sequence effects).

Before the ratings were made, participants answered questions about their demographic characteristics (native language, nationality, age, gender, educational level). If a participant did not meet the inclusion criteria (i.e., indicating an age under 18 or over 65, or if they were not a native speaker of English), they were excluded from further consideration by Qualtrics routines. Thereafter, participants were instructed in detail about the forthcoming task requirements. Task comprehension was checked following these instructions with two short questions that must be answered correctly in order to continue (see *Study Tasks - Comprehension Check*). Further exclusion criteria were established during or following participation (see *Data Integrity Checks*). The median response time was 36.5 min (IQR = 24.37). Participants who took longer than 12 h to complete the study and those who voluntarily terminated the study were not used for data analyses.

### Study tasks

#### Rating tasks

For the VAL ratings, participants were asked to rate each word with respect to the trait’s emotional meaning by placing a cross below *+1, +2,* or *+3* for each word to indicate its degree of positivity for them personally, or a cross below *–1, –2*, or *–3* for any word to indicate its degree of negativity. The scale end-points *(+3, –3*) were labeled *Extremely Positive* or *Extremely Negative*, respectively, and the *0* was labeled *Neutral*. This seven-point Likert scale has been commonly used in word-rating studies (e.g., Britz et al., 2019; Ric et al., 2013; Võ et al., 2006), and should reflect the extent of emotional content that participants associated with this trait.

For the SOC ratings, participants were asked to indicate the degree of social desirability they associated with each word. They were informed that personality traits can be socially desirable to varying degrees, and that they more or less correspond to society’s expectations. They were asked to place a cross below *+1, +2*, or *+3*) for any word they considered to be a desirable trait in society and to place a cross below *–1, –2*, or *–3* for those words they considered to be an undesirable trait. The scale endpoints were labeled *Extremely Socially Desirable* or *Extremely Socially Undesirable*, and the *0* was labeled *Neutral*. The numeric value on the provided seven-point Likert scale should reflect the extent of social desirability that participants associated with this trait.

For the OBS ratings, participants were asked to rate each word for how observable the trait was to other people. Participants were informed that personality traits can be visible to an observer to varying degrees, i.e., some traits are easy to observe, whereas other traits are difficult to observe. Any trait that they considered to be easy to observe should be indicated by placing a cross below a higher value on the four-point Likert scale that ranged from *1* (*Extremely difficult to observe*) to *4* (*Extremely easy to observe*). Although previous literature has mainly used nine-point Likert scales (John & Robins, 1993; Moran et al., 2011), seven-point Likert scales (Quadflieg et al., 2014), or a categorical scale (high vs. low observability; Human & Biesanz, 2011), a four-point Likert scale was chosen since a more fine-grained differentiation between the answer alternatives was not expected while still allowing for sufficient variability in the ratings.

For all of the ratings, participants could also indicate “I don’t know the meaning of the word.” Further, they were informed that ten non-words were included to check for participants’ concentration during the task, and when a non-word was presented, they should click, “I don’t know the meaning of the word.” In a post hoc integrity check, it was determined to what extent participants had correctly identified the non-words (see *Data Integrity Checks *and Supplements 2).

#### Comprehension checks

Before the task was completed, two multiple choice questions were administered in order to check participants’ comprehension of the instructions. The first question was identical in all rating conditions (VAL, SOC, and OBS). It asked participants to indicate by which criteria they were supposed to make their evaluations. The response options were (a) “the negativity or positivity of character traits”, (b) “society’s desirability criteria of character traits”, and (c) “the observability of character traits.” In the VAL condition (i.e., when the valence of the word was to be rated), the second question asked about the valence of the word “violent,” using the response options (a) “a positive trait of a person”, (b) “a negative trait of a person”, or (c) “neither a positive nor a negative trait of a person”. In the SOC condition (i.e., when social desirability was rated), participants had to indicate whether a socially desirable trait was (a) “an attribute/characteristic that is appreciated in society”, (b) “an attribute/characteristic that is frowned upon in society”, or (c) “an attribute/characteristic that is neither appreciated nor frowned upon in society.” If both multiple-choice questions were answered correctly, participants proceeded to rating the trait adjectives. For the OBS ratings, only the first question had to be answered correctly. For the second question, using a word example was not applicable for the observability ratings, because there was no objectifiable correct or incorrect answer in the earlier literature or previous findings.

#### Data integrity checks

The integrity of the data was checked (a) to see whether the participants recognized the non-words and (b) to determine the variability in the ratings. Participants who did not identify at least five of the non-words and those who pressed the same rating category more than 30 times in a row were excluded from the analyses.

### Participants and analysis samples

#### Participants

The final sample comprised 822 participants (see Table 1 and Fig. 1, Supplements 4 gives an overview of the dropouts), including 472 (57.42%) females and 350 (42.58%) males who were evenly distributed across the rating conditions (χ^2^(3,822) = 0.21, *p* = .976). Participants’ mean age was 44.72 (*SD* = 13.44) years and ranged from 18 to 65 (females’ mean age: 47.75, *SD* = 13.37; males’ mean age: 40.62, *SD* = 12.39), with the female participants being significantly older (*ΔM* = – 7.12, 95% CI [– 8.92, – 5.33], *t*(820) = – 7.79, *p* < .001; see Table 1). For group comparisons on age, participants were divided into three age groups: 18–34 years (*younger*, *n* = 228), 35–49 years (*middle-aged*, *n* = 260), and 50–65 years (*older*, *n* = 324). Participants’ mean years of education was 15.09 (*SD* = 4.55) years (see Table 1), which did not significantly differ among the three age groups (*F*(2,806) = 1.57, *MSE* = 20.77, *p* = .208, ${\hat{\eta}}_G^2$ = 0.004) but did significantly differ between the two genders (*F*(1,806) = 4.07, *MSE* = 20.77, *p* = .044, ${\hat{\eta}}_G^2$= 0.005) although with a small effect size.

**Table 1:** Participant characteristics

Condition	*N* Total	*N* Females	Females (%)	Age		Age females		Age males		Education (in years)	
				Mean	SD	Mean	SD	Mean	SD	Mean	SD
SO1	203	114	56.16	44.61	14.00	47.18	14.18	41.30	13.11	15.20	4.41
SO2	204	117	57.35	45.42	13.62	48.18	14.08	41.71	12.11	15.21	5.42
VO1	206	120	58.25	44.87	13.02	49.10	11.94	38.98	12.21	15.07	3.95
VO2	209	121	57.89	43.98	13.12	46.53	13.28	40.48	12.13	14.88	4.31
Total	822	472	57.42	44.72	13.44	47.75	13.37	40.62	12.39	15.09	4.52

SO1 = SOC and OBS ratings of word list 1; SO2 = SOC and OBS ratings of word list 2; VO1 = VAL and OBS ratings of word list 1; VO2 = VAL and OBS ratings of word list2; *N* Total = Total number of participants in condition; *N* Females = Number of female participants in condition; Females (%) = Percentage of female participants in condition

#### Samples for analysis on word level

Because the 500 words were divided into Word List 1 and Word List 2 with 250 adjectives in each list and rating condition (see Fig. 1), each word was rated by approximately 400 participants. The calculations for the trait database were conducted at the word level, i.e., the ratings were compared across all participants who rated a word. Calculations were conducted separately for each rating condition, resulting in 203 ratings for SOC and OBS for Word List 1 and 204 ratings for SOC and OBS for Word List 2. The calculations for VAL and OBS were based on 206 ratings for Word List 1 and 209 ratings for Word List 2 (see Fig. 1).

#### Samples for analysis on group level

Additional analyses were conducted at the group level across the 500 trait words in order to further identify characteristics of the SOC, VAL, and OBS ratings. For these analyses, participants who rated Word List 1 and Word List 2 were merged, resulting in 407 participants who rated SOC and 415 participants who rated VAL. Because OBS was rated in each condition (both SOC and VAL and Word List 1 and Word List 2), averaging the OBS ratings resulted in a total of 822 OBS ratings. The OBS ratings in the SOC and VAL conditions of Word List 1 (*r* = 0.98, 95% CI [0.98, 0.99], *t*(248) = 85.81, *p* < 0.001) and 2 (*r* = 0.98, 95% CI [0.97, 0.98], *t*(248) = 70.02, *p* < 0.001) were found to be significantly correlated, indicating strong consistency in the OBS ratings.

### Data analysis and results

R [Version 4.1.0; R Core Team, 2021] was used for all of the analyses.

The following section includes a description of the content of the ELoT Database. Furthermore, we describe internal consistencies, the characteristics, response distributions, and the consistencies of the VAL, SOC, and OBS ratings, and the age and gender effects. Finally, we describe the associations between (a) the VAL, SOC, and OBS ratings and (b) the psycholinguistic variables, and (c) their associations with the variables in the other databases.

#### Database

The database contains 500 words with normative data on valence, social desirability, and observability ratings and on psycholinguistic variables provided by at least 200 participants. The trait database was organized into five database tables (see Table 2 for an overview). Each table contains two pages. The first one presents the actual data from the database (*Database*), and the second one provides a detailed description of the data columns (*Labels*). Database table 1 additionally includes participants’ instructions for completing the VAL, SOC, and OBS ratings. The complete ELoT can be found at https://osf.io/23hc8/?view_only=46298b4d67d54fe58d5491f1b938e31f. The link https://personality-traits.eu includes a tool that allows researchers to select stimulus material from the ELoT based on various attributes that might be of interest or that might need to be controlled in experimental studies.

**Table 2:** Structure of the ELoT database

Database tables	Column	Description
1 Ratings	*N*	Number of participants in this condition (rating and word list)
	I DON’T KNOW MEANING	Number of participants who indicated they don’t know the meaning of this word
	MEAN	Rating mean across participants
	SD	Standard deviation across participants
	RESPONSE DISTRIBUTION	Rating scale usage (e.g., number of times the response options of the 7-point Likert for the SOC & VAL ratings and 4-point Likert scale for the OBS ratings were used to rate a word)
2 Rating effects	*N*	Number of participants who rated VAL or SOC ratings
	ABS.DIFFERENCE VAL-SOC	Mean difference between the VAL and SOC ratings across participants
	T-VALUE (DIFF VAL-SOC)	T-value of independent sample *t* test between VAL and SOC per word
	EFFECT SIZE (DIFF VAL-SOC)	Cohen’s d effect size for independent samples (d=t/$\sqrt{\left({SD}_1^2+{SD}_2^2\right)/2}$)
	COR	Correlation between two ratings*
3 Gender effects	*N*	Number of participants with this gender
	MEAN	Mean ratings across participants with this gender
	SD	Standard deviation across participants with this gender
	COR VAL-OBS FEMALE	Correlation between VAL and OBS ratings across female participants
	COR VAL-OBS MALE	Correlation between VAL and OBS ratings across male participants
	COR SOC-OBS FEMALE	Correlation between SOC and OBS ratings across female participants
	COR SOC-OBS MALE	Correlation between SOC and OBS ratings across male participants
4 Age effects	*N*	Number of participants in the age group
	MEAN	Mean ratings across participants in this age group
	SD	Standard deviation across participants in this age group
5 Psycho-linguistics	SYLLABLES	Number of syllables
	LETTERS	Number of letters
	CELEX_FREQUENCY_LOG10	Logarithmized word frequency per 1 million words of the Celex database to base 10
	SUBTLEX_FREQUENCY_ZIPF	Logarithmized word frequency per 1 billion words of the SUBTLEX database to base 10.
	FREQUENCY AVAILABLE	2=SUBTLEX and CELEX frequency available; 1=SUBTLEX or CELEX frequency available; 0=no frequency available

SD = Standard deviation; * Since paired data is required to calculate the correlations on word level, correlations could only be calculated for each word within a rating condition (VAL&OBS, SOC&OBS). Zipf Frequency values were taken from the SUBTLEX-US database and are freely accessible online (https://osf.io/7wx25/).

#### Internal consistencies

The internal consistency of the SOC, VAL, and OBS rating conditions was assessed by calculating split-half reliability for each condition and for which the datasets were randomly divided into two halves. All of the ratings conditions had a high degree of internal consistency (Word List 1, SOC: *r* = .96, VAL: *r* = .90, and OBS: *r* = .99; Word List 2, SOC: *r* = .94, VAL: *r* = .93, and OBS: *r* = .99).

Further, correlations among the VAL, SOC, and OBS ratings between female and male participants revealed a high consistency in the ratings across all of the 500 trait adjectives (VAL: *r* = .99, 95% CI [0.99, 0.99], *t*(498) = 162.22, *p* < .001; SOC: *r* = .99, 95% CI [0.99, 0.99], *t*(498) = 182.70, *p* < .001; OBS: *r* = .95, 95% CI [0.94, 0.96], *t*(498) = 69.10, *p* < .001).

#### Characteristics of the VAL, SOC, and OBS ratings

Means, standard deviations, and ranges over all of the 500 words and all of the participants were calculated for the VAL, SOC, and OBS ratings (*Ratings*, column VAL_MEAN, SOC_MEAN and OBS_MEAN). Across all 500 words, the VAL ratings had a mean of .20 (*SD* = 1.55; range_min-max_ = – 2.80–2.58). Similarly, the SOC ratings had a mean of .20 (*SD* = 1.68; range_min-max_ = – 2.70–2.69). OBS was rated with a mean of 2.91 (*SD* = 0.34; range_min-max_ = 1.98–3.76). The ten words with the highest and the ten words with the lowest mean VAL, SOC, and OBS ratings are also shown in Table 3 (Supplements 5 additionally shows a Table including the ten words with highest and lowest VAL, SOC, and OBS ratings separately for female and male participants).

**Table 3:** Top ten trait adjectives with highest and lowest VAL, SOC and OBS ratings

VAL			SOC			OBS		
Words	Mean	SD	Words	Mean	SD	Words	Mean	SD
loving	2.58	0.76	trustworthy	2.69	0.73	loud	3.76	0.59
honest	2.58	0.67	hardworking	2.56	0.89	pretty	3.71	0.57
trustworthy	2.49	0.82	helpful	2.55	0.72	attractive	3.70	0.53
compassionate	2.45	0.76	respectable	2.51	0.71	talkative	3.70	0.56
reliable	2.43	0.76	kind	2.50	0.88	beautiful	3.67	0.59
traitorous	– 2.62	0.77	tyrannical	– 2.52	1.07	lonely	2.16	1.00
hateful	– 2.64	0.64	traitorous	– 2.54	1.07	traitorous	2.14	1.04
corrupt	– 2.67	0.68	malicious	– 2.55	0.88	sly	2.14	0.89
cruel	– 2.67	0.67	violent	– 2.66	0.88	underhand	2.11	0.92
racist	– 2.8	0.58	racist	– 2.7	0.94	unfaithful	1.98	1.03

SD = Standard deviation; VAL & SOC ratings conducted on seven-point Likert ranging from – 3 to 3; OBS ratings conducted on four-point Likert scale ranging from 1 to 4; values taken from database 1 (*Ratings*); for the top ten trait adjectives of females and males see Supplements 5

#### Age and gender differences in the VAL, SOC, and OBS ratings

To assess whether the VAL, SOC, and OBS mean ratings differed between the three age groups and between males and females, three 3 x 2 factorial ANOVAS were conducted separately for the VAL, SOC, and OBS ratings with age (younger, middle-aged, older) and gender (male, female) as the between-participant factors and the mean ratings of all 500 words across all participants as the dependent variable. The values used in these analyses refer to the mean ratings in database 4 *Age effects* (e.g., columns VAL AGE 18–34 MEAN, VAL AGE 35–49 MEAN, VAL AGE 50–65 MEAN). Please note, that the age groups of database 4 were further split into female and male participants. In addition to the generalized eta squared effect sizes (${\hat{\eta}}_G^2$) with respective confidence intervals, the mean square of error (MSE) is reported as the recommended effect size for analyses of variance using categorical independent variables (such as age groups or gender; Olejnik & Algina, 2003)^1^. The ANOVA on the VAL ratings revealed neither a significant main effect for age (*F*(1,2,996) = 0.01, *MSE* = 2.4, *p* = .93, ${\hat{\eta}}_G^2$ = 0.00) nor gender (*F*(1,2,996) = 2.49, *MSE* = 2.4, *p* =.12, ${\hat{\eta}}_G^2$ = 0.001). The interaction was also non-significant (*F*(1,2,996) = 0.94, *MSE* = 2.4, *p* = .33, ${\hat{\eta}}_G^2$ = 0.00). Similarly, the ANOVA on the SOC ratings revealed neither a significant main effect for age (*F*(1,2,996) = 1.76, *MSE* = 2.86, *p* = .18, ${\hat{\eta}}_G^2$ = 0.001) nor gender (*F*(1,2,996) = 0.87, *MSE* = 2.86, *p* = .35, ${\hat{\eta}}_G^2$ = 0.00) nor the interaction (*F*(1,2,996) = 2.11, *MSE* = 2.86, *p* = .153, ${\hat{\eta}}_G^2$ = 0.001). Lastly, only the ANOVA on the OBS ratings revealed a significant main effect for both age, (*F*(1,2,996) = 33.37, *MSE* = 0.12, *p* < .001, ${\hat{\eta}}_G^2$ = 0.011) and gender (*F*(1,2,996) = 31.16, *MSE* = 0.12, *p* < .001, ${\hat{\eta}}_G^2$ = 0.01) but the interaction was non-significant, (*F*(1,2,996) = 1.58, *MSE* = 0.12, *p* = .21, ${\hat{\eta}}_G^2$ = 0.001). Post hoc tests revealed significantly different OBS means among all three of the age groups. Table 4 shows the means, standard deviations, *p* values, and effect sizes for the respective analyses.

**Table 4:** VAL, SOC, and OBS rating means and standard deviations of positive vs. negative words across females and males as well as younger, middle-aged, and older participants

		Female		Male						Younger		Middle-aged		Older					
		*Mean*	*SD*	*Mean*	*SD*	*p*	${\hat{\eta}}_G^2$	90%	CI	*Mean*	*SD*	*Mean*	*SD*	*Mean*	*SD*	*p*	${\hat{\eta}}_G^2$	90%	CI
VAL	total	0.19	1.01	0.21	1.05	.115	0.001	0.000	0.003	0.20	1.07	0.19	1.03	0.21	0.99	.927	0.000	0.000	.001
	high	1.50	0.65	1.42	0.62					1.42	0.63	1.46	0.62	1.51	0.66				
	low	– 1.39	0.70	– 1.32	0.63					– 1.34	0.63	– 1.36	0.65	– 1.38	0.73				
SOC	total	0.18	1.11	0.23	1.19	.351	0.000	0.000	0.002	0.26	1.22	0.21	1.18	0.16	1.08	.184	0.001	0.000	0.003
	high	1.66	0.66	1.57	0.67					1.59	0.68	1.62	0.67	1.66	0.66				
	low	– 1.56	0.70	– 1.4	0.63					– 1.36	0.62	– 1.48	0.68	– 1.57	0.69				
OBS	total	2.85	0.85	2.97	0.88	< .001	0.010	0.005	0.017	2.63	0.85	2.92	0.85	2.86	0.85	< .001	0.011	0.006	0.018
	high	2.97	0.30	3.02	0.27					3.01	0.27	2.98	0.28	2.94	0.27				
	low	2.31	0.16	2.37	0.10					2.22	0.17	2.36	0.11	2.35	0.14				

*P* = p-value of effect of gender, resp. age on ratings; ${\hat{\eta}}_G^2$ = effect size generalized eta-squared of effect of gender, resp. age on ratings; 90%CI = 90% Confidence interval of effect size; *SD* = Standard Deviation; *Younger* = Age 18–34 years; *Middle-aged* = Age 35–49 years; *Older* = Age 50–65 years; values taken from database 1 (*Ratings*), 3 (*Gender effects*) and 4 (*Age effects*)

#### Response distributions and consistencies of VAL, SOC, and OBS

First, the percent use of the individual rating scale response options (i.e., seven points for VAL and SOC ratings and four points regarding OBS rating) was calculated, using the number of times a rating scale point option was indicated over all words and participants with regard to VAL, SOC, and OBS rating, respectively (see Table 5). These data were calculated based on database 1 (*Ratings*, columns RESPONSE DISTRIBUTION VAL; RESPONSE DISTRIBUTION SOC and RESPONSE DISTRIBUTION OBS). The response option with the highest percent use of the rating scale response options in the VAL ratings was *0* (18.45%). Regarding the VAL ratings with a mean above and below 0, the response options *+2* (26.92%) and *–1* (29.26%) had the highest percent usage. For the SOC total ratings, a different pattern emerged, with the response option *+3* (17.64%) having the highest percent usage. For the positive SOC ratings (mean above *0*) and the negative SOC ratings (mean below *0*), the response options *+3* (28.75%) and *–3* (31.64%) were used most often, indicating a bimodal distribution of responses. Lastly, the response option +3 (37.89%) was used most often on the four-point Likert scale for the OBS ratings.

**Table 5:** Percent use of rating scale points for VAL, SOC, and OBS ratings

		Percent use of rating scale point							
	Scale points	– 3	– 2	– 1	0	1	2	3	4
Ratings									
VAL total		10.43	10.79	14.34	18.45	16.54	15.57	13.88	
VAL positive		0.46	0.75	2.57	18.12	26.87	26.92	24.31	
VAL negative		23.06	23.51	29.26	18.86	3.45	1.20	0.66	
SOC total		14.41	11.42	11.60	14.28	14.60	16.04	17.64	
SOC positive		0.90	1.25	2.88	15.71	23.99	26.52	28.75	
SOC negative		31.64	25.25	22.26	13.46	3.84	1.61	1.93	
OBS total						7.68	23.23	37.89	31.20

VAL positive = VAL ratings with mean > 0; VAL negative = VAL ratings with mean < 0; SOC positive = SOC ratings with mean > 0; SOC negative = SOC ratings with mean < 0; values taken from database 1 (*Ratings*)

**Fig. 2:**
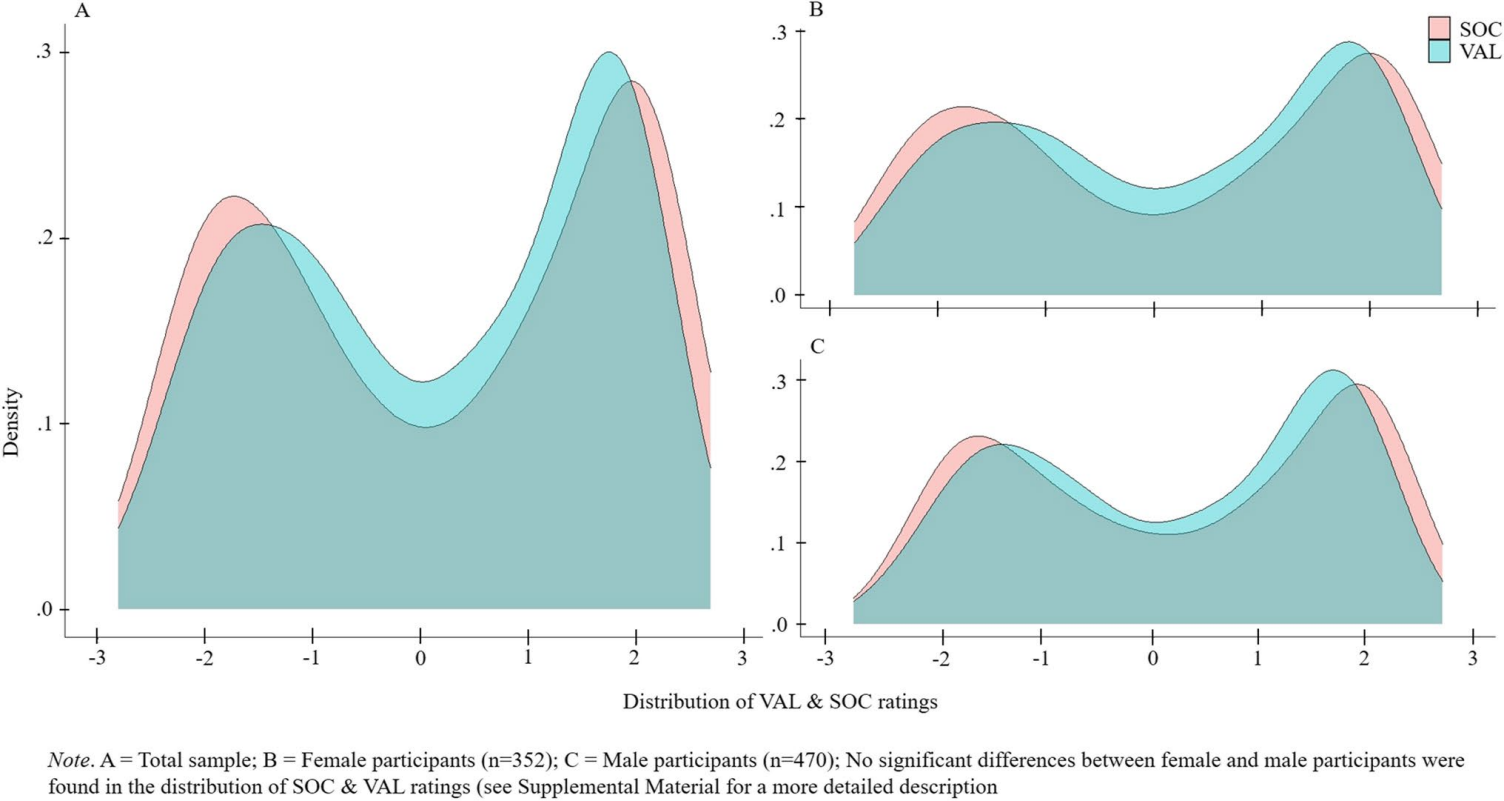
Rating distribution of VAL, SOC & OBS ratings across all participants (**A**) and separately for female (**B**) and male participants (**C**)

Second, the distribution of the VAL and SOC rating means across the total sample as well as separately for female and male participants was inspected and is displayed in a density distribution (see Fig. 2; referring to database 1 *Ratings*, column VAL_MEAN, SOC_MEAN). Gender and age group differences in the response distribution of VAL, SOC and OBS ratings were investigated using ANOVAS (see Supplements 7). Figure 2 shows a bimodal distribution of the VAL and SOC rating means with a higher concentration of ratings in the mid-negative and mid-positive ranges and with fewer ratings in the neutral range. The VAL ratings were, however, somewhat more densely distributed than the SOC ratings. Figure 2 also shows that the distribution of VAL and SOC rating means did not differ between the female and male participants, as was confirmed by the non-significant ANOVAs for gender in the VAL, SOC, and OBS ratings (in addition, no significant differences were found between the three age groups regarding the response distribution of VAL, SOC, and OBS ratings; see Supplements 7 for a more detailed description).

**Fig. 3:**
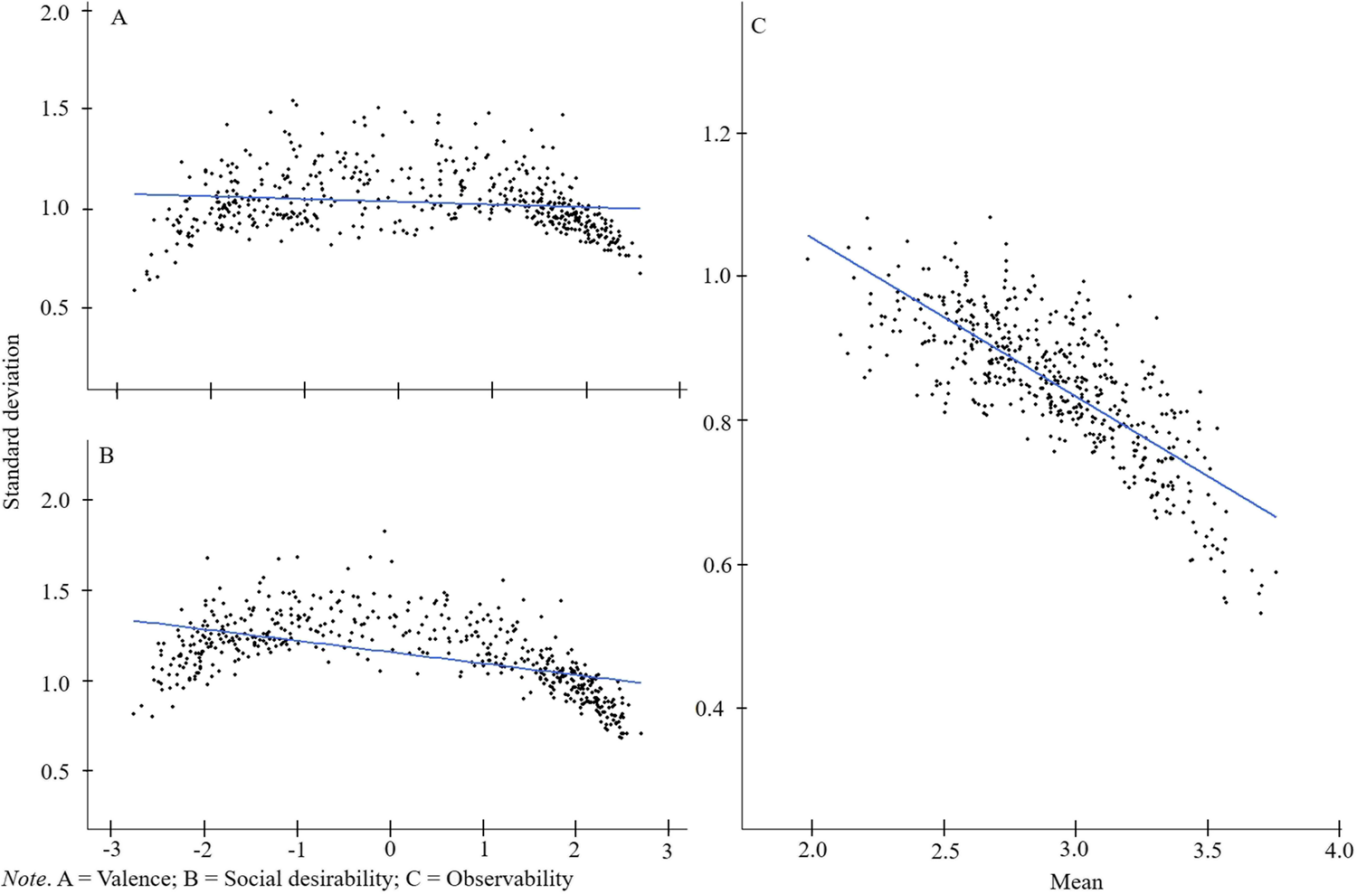
Association between rating means  and standard deviation for SOC (**A**), VAL (**B**), and OBS (**C**) ratings

Third, to further inspect the consistency of rating scale usage regarding the rating scale end- versus scale midpoints the VAL, SOC, and OBS rating means and standard deviations (referring to database 1 *Ratings*, column VAL_MEAN, SOC_MEAN, OBS_MEAN, VAL_SD, SOC_SD, and OBS_SD) were correlated using Pearson’s *r* and displayed in a scatterplot. Significant negative correlations between means and standard deviations were found for all of the ratings (VAL: *r* = – .14, 95% CI [– 0.22, – 0.05], *t*(498) = – 3.08, *p* = .002; SOC: *r* = – .53, 95% CI [– 0.59, – 0.47], *t*(498) = – 14.10, *p* < .001; OBS: *r* = – .77, 95% CI [– 0.81, – 0.74], *t*(498) = – 27.19, *p* < .001). In addition, a curvilinear relationship was found between the means and standard deviations for the SOC and VAL ratings, illustrating that the ratings were more widely dispersed around the scale midpoints and with a smaller dispersion at the scale endpoints (see Fig. 3a, b). By contrast, the OBS means and standard deviations of the ratings show a negative linear association, with smaller standard deviations for the larger mean ratings (see Fig. 3c).

#### Contrasting divergent VAL and SOC ratings

To analyze possible differences between VAL and SOC ratings the focus was put on those words for which the VAL and SOC rating difference emerged from a positive rating in one rating condition and a negative rating in the other rating condition. Note that, so far, the rating differences shown in the database 2 (*Rating effects*, e.g., column ABS.DIFFERENCE VAL-SOC) are presented in absolute numbers to show the extent of the deviations between the rating scales. Therefore, for example, an absolute difference of 2 of a word rated as *+3* positive and as *+1* social desirable would be the same, as for a word rated as *+1* positive in valence and as –*1* social desirable. However, words whose rating +/– sign differs between the rating conditions may be more different in terms of the dimensions to be assessed than words whose rating +/– sign does not differ. Eight divergent words were identified and are shown in Table 6 with the corresponding mean, standard deviation, and the absolute difference between VAL and SOC ratings. The trait adjectives *guarded*, *laconic*, *cheeky*, *controlled*, *eccentric*, *introverted*, and *silent* were rated negative on social desirability, but they were rated with a mild positive valence. The largest difference between the SOC and VAL ratings was for the word *eccentric*, which was followed by *introverted*. A positive SOC and negative VAL rating was found only for the word *indulgent*.

**Table 6:** Trait adjectives with divergent VAL and SOC ratings

Trait adjective	VAL Mean	VAL SD	SOC Mean	SOC SD	Diff VAL-SOC
eccentric	0.31	1.04	– 0.30	1.29	0.61
introverted	0.06	0.89	– 0.54	1.29	0.61
laconic	0.04	1.20	– 0.36	1.34	0.40
silent	0.10	0.87	– 0.23	1.11	0.33
controlled	0.08	1.49	– 0.06	1.83	0.14
cheeky	0.11	1.16	– 0.02	1.23	0.13
guarded	0.02	0.94	– 0.10	1.05	0.12
indulgent	– 0.06	1.14	0.14	1.42	– 0.20

VAL Mean = Mean of the valence ratings, SOC Mean = Mean of the social desirability ratings, Diff VAL-SOC = Absolute difference between VAL and SOC rating means; Values taken from database 1 (*Ratings*)

#### Associations among VAL, SOC, and OBS ratings and psycholinguistics

Pearson’s *r* correlations among VAL, SOC and OBS ratings and the psycholinguistic variables word length and frequency (derived from CELEX, SUBTLEX) were calculated to inspect the validity of the ratings. These calculations refer to the values in database 1 (*Ratings*, columns VAL_MEAN, SOC_MEAN and OBS_MEAN) and database 5 (*Psycholinguistics*, columns LETTER, CELEX_FREQUENCY_LOG10 and SUBTLEX_FREQUENCY_ZIPF). Positive associations between the ratings and their word frequencies were found which is in line with previous findings (e.g., Gilet et al., 2012). Moreover, negative although non-significant associations between the ratings and word length were found. The correlations between the ratings and psycholinguistic variables are shown in Table 7.

**Table 7:** Associations among VAL, SOC, and OBS ratings, and the ANEW and EMOTE as well as psycholinguistic variables

Variable	1	2	3	4	5	6	7	8
1 VAL								
2 SOC	.99***							
3 OBS	.23***	.24***						
4 ANEW VAL	.92***	.91***	.31***					
5 EMOTE VAL	.96***	.95***	.21***	.92***				
6 EMOTE SOC	.95***	.95***	.19***	.89***	.96***			
7 Celex freq	.23***	.24***	.22***	.26**	.21***	.18**		
8 SUBTLEX freq	.21***	.22***	.30***	.16*	.19***	.17**	.81***	
9 LETTERS	– .05	– .04	– .12**	.06	.01	.04	– .32***	– .46***

VAL = Valence ratings of ELoT database, SOC = Social desirability ratings of ELoT database, OBS = Observability ratings of ELoT database, ANEW VAL = Valence ratings of ANEW database (Bradley & Lang, 1999), EMOTE VAL = Valence ratings of EMOTE database (Grühn, 2016), EMOTE SOC = Desirability ratings of EMOTE database (Grühn, 2016), Celex freq = log10 frequency per million words from the Celex database (Baayen et al., 1993), SUBTLEX freq = Zipf frequency (log10 per billion words) from the SUBTLEX-US database (https://osf.io/7wx25/), *p < .05, **p < .01,***p < .001

#### Associations among VAL, SOC, and OBS ratings

**Fig. 4:**
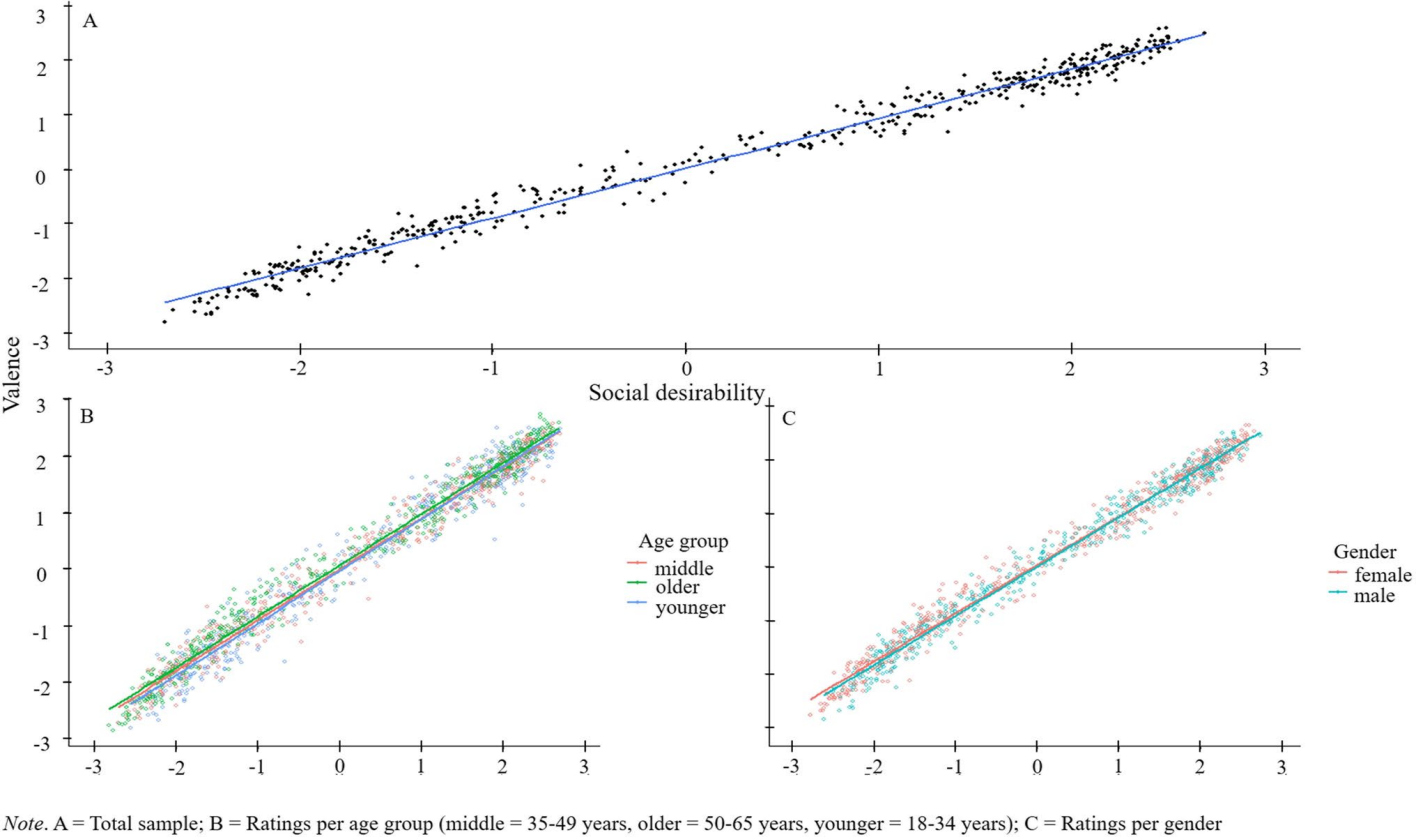
Association between valence and social desirability ratings across all participants (**A**) and separately for age (**B**), and gender (**C**)

To investigate the association among the rating dimensions, the mean VAL, SOC, and OBS ratings over all subjects across all 500 words per rating condition Pearson’s *r* correlations were calculated (see database 1 *Ratings*, columns VAL_MEAN, SOC_MEAN and OBS_MEAN). The results indicated strong associations between the VAL and the SOC ratings (*r* = .99, 95% CI [0.99, 0.99], *t*(498) = 182.82, *p* < .001) (Fig. 4a), whereas the OBS ratings were only moderately associated with both VAL (*r* = .23, 95% CI [.15, .32], *t*(498) = 5.38, *p* < .001) and SOC ratings (*r* = .24, 95% CI [0.16, 0.32], *t*(498) = 5.52, *p* < .001). The correlations among the VAL, SOC, and OBS ratings are also shown in Table 7.

The same correlation analyses were also conducted separately for males and females (see Fig. 4c). Again, the VAL and SOC ratings revealed similarly strong associations (females: *r* = .99, 95% CI [0.99, 0.99], *t*(498) = 158.71, *p* < .001, males: *r* = .99, 95% CI [0.99, 0.99], *t*(498) = 163.54, *p* < .001). Of note, the male participants had a higher association between the VAL and the OBS ratings (*r* = .19, 95% CI [.10, .27], *t*(498) = 4.22, *p* < .001), and the SOC and OBS ratings (*r* = .19, 95% CI [0.10, 0.27], *t*(498) = 4.28, *p* < .001) than the female participants (VAL & OBS: *r* = .15, 95% CI [0.06, 0.23], *t*(498) = 3.30, *p* = .001; SOC & OBS: *r* = .16, 95% CI [0.08, 0.25], *t*(498) = 3.70, *p* = .001), suggesting that males perceived the traits as more positive and socially desirable when these traits were more observable. Post hoc independent samples *t* tests showed a significant difference between the female and male participants in the correlations between SOC and OBS per word for Word List 1 (*t*(498) = 2.63, *p* = .008) and Word List 2 (*t*(498) = – 2.84, *p* = .004). The difference between males and females in the VAL and OBS correlations, however, was non-significant (Word List 1: *t*(498)= 0.240, *p* = .81; Word List 2: *t*(498) = – 1.58, *p* = .114).

Lastly, the correlations among VAL, SOC, and OBS were assessed across the three age groups. The VAL and SOC ratings were strongly associated in all three of the age groups (see Fig. 4b) (younger: *r* = .98, 95% CI [0.98, 0.99], *t *(498) = 118.28, *p* < .001; middle-aged: *r* = .99, 95% CI [0.99, 0.99], *t*(498) = 148.61, *p* < .001; older: *r* = .99, 95% CI [0.99, 0.99], *t*(498) = 152.39, *p* < .001). The strongest association between VAL and OBS was in the middle-aged participants (*r* = .22, 95% CI [0.14, 0.30], *t*(498) = 5.07, *p* < .001) who were followed by the older (*r* = .19, 95% CI [0.11, 0.28], *t*(498) = 4.43, *p* < .001) and then the younger (*r* = .17, 95% CI [0.08, 0.25], *t*(498) = 3.82, *p* < .001) participants. A similar pattern occurred in the associations between SOC and OBS. Again, the strongest association was in the middle-aged age group (*r* = .24, 95% CI [0.15, 0.32], *t*(498) = 5.45, *p* < .001), which was followed by the older (*r* = .20, 95% CI [0.12, 0.28], *t*(498) = 4.59, *p* < .001) and then the younger participants (*r* = .19, 95% CI [0.11, 0.28], *t*(498) = 4.39, *p* < .001).

#### Associations among VAL, SOC, and OBS ratings and other databases

The validity of the ratings was further examined by inspecting the overlap of the ratings obtained in the current study with ratings of the ANEW (Bradley & Lang, 1999) and EMOTE (Grühn, 2016) database. The ANEW is a database comprised of 1034 words of which 202 words overlapped with the ELoT database. The words in the ANEW were rated on the dimensions pleasure (valence), arousal, and dominance/control (Bradley & Lang, 1999). The EMOTE database contains 958 adjectives rated on several dimensions such as imagery, concreteness, familiarity, as well as valence and desirability amongst others (Grühn, 2016). A total of 340 words of the EMOTE overlapped with the trait adjectives of the ELoT. The associations between the ELoT and the other databases were calculated using Pearson’s *r*. The values used for these analyses refer to ELoT database 1 (*Ratings*, columns VAL_MEAN and SOC_MEAN) and to the valence ratings of the ANEW (ANEW VAL) and the valence and desirability ratings of the EMOTE (EMOTE VAL & SOC). The ELoT VAL ratings showed a high correlation with both, the VAL ratings of the ANEW (*r* = .92, 95% CI [0.89, 0.94], *t*(200) = 32.27, *p* < .001), and the valence ratings of the EMOTE (*r* = .96, 95% CI [0.95, 0.97], *t*(338) = 63.64, *p* < .001) database. Further, the ELoT SOC ratings were highly correlated with the EMOTE SOC ratings (*r* = .95, 95% CI [0.94, 0.96], *t*(338) = 55.89, *p* < .001). The correlations between the different databases are displayed in Table 7.

## Discussion

The aim of the present study was to create a database of English words that could be used for manipulation and control of the stimuli in experimental research. The database that was created includes valence, social desirability, and observability ratings for each of 500 adjectives that depict personality traits, along with various psycholinguistic variables. The most important characteristics of the database and its special features with regard to scale usage, associations among ratings, and group differences are as follows:

### Response distributions and consistencies of VAL, SOC, and OBS

Similar to Anderson (1968) and the German trait word database (Britz et al., 2019), both the VAL and the SOC rating means had a bimodal distribution, which indicates that most of the words were rated either positively (respectively socially desirable) or negatively (respectively socially undesirable), and fewer words were rated as neutral (see Fig. 2; Anderson, 1968; Britz et al., 2019). Figure 3a and b additionally show that the words at the end-points of the rating scales (i.e., with very positive or very negative rating means) were rated more consistently than the words with a neutral rating mean. For the OBS ratings, there was a bell-shaped distribution. There was a negative linear relationship between the OBS means and standard deviations, indicating that words with a higher mean rating were rated more consistently (see Fig. 3c). The bimodal distribution of the VAL and SOC rating means shown in Fig. 2 is noteworthy because it appears to contradict the use of the rating-scale points shown in Table 5. It should be noted, however, that Fig. 2 depicts the positive and negative rating means in a density distribution, which is not the same as the use of the rating-scale points shown in Table 5. When considering the percent use of each point on the seven-point Likert scale for the VAL and SOC ratings across all of the words, it might appear that the neutral point (*0*) was used as often as the positive and negative rating scale points. However, when considering the percent use of the rating-scale points separately for the words with positive or negative rating means, it becomes clear that the end points of the rating scale were used more frequently than the neutral point as the bimodal distribution in Fig. 2 shows. Based on Table 5, it seems that the neutral rating point is the scale point that is used most frequently, however, it does not result in a high number of words with an average neutral qualification. Rather, both the rating scale use separately for positive and negative words shown in Table 5 and the scatterplots shown in Fig. 3 suggest that some of the neutral ratings arose from averaging the positive and negative ratings. Many of the zero ratings are outweighed by either highly positive or highly negative ratings, yielding a bimodal distribution of the average ratings. Taken together, these findings indicate that different individuals might have perceived the neutral words differently. It is questionable, therefore, whether there are some words that are truly neutral in valence. The valence or perceived social desirability that a word has for a given individual might be influenced by numerous factors, such as a person’s cultural background (Lalwani et al., 2009), gender (e.g., Dill & Thill, 2007; Montefinese et al., 2014), age (Charles et al., 2016), or simply the individual’s own life experiences. Researchers should thus consider whether a word that appears to be neutral based on mean ratings might still be very positive or very negative for an individual. We suggest, therefore, that the dispersion of the words (e.g., their standard deviation) should be taken into account when neutrally valenced stimuli are used in research; e.g., researchers might consider using only neutral words with a small standard deviation (e.g., < 2.0).

### Age and gender differences in the VAL, SOC, and OBS ratings

The current study also investigated rating differences depending on the participants’ age or gender. On a general note, group effects have not been investigated frequently in word databases, since previous studies largely focused on extensive databases of adjectives and/or nouns with ratings of comparably fewer participants. Most prior studies have analyzed age and gender differences only in valence ratings (e.g.,Gilet et al., 2012 ; Grühn & Smith, 2008 ; Söderholm et al., 2013), and the effects of age and gender on social desirability and observability ratings are limited. Because the current study was conducted via an online platform, a large sample was acquired, which enabled us to compare differences in VAL, SOC, and OBS ratings for the three age groups and for male and female participants.

Women and men are often exposed to different socialization processes, resulting in the internalization of different values into their self-concept (Ampofo, 2001). Consequently, women and men may have different ideas about which character traits are desirable (e.g., Dill & Thill, 2007). Generally, males had higher mean ratings than females in all three of the rating dimensions. A significant effect of gender was, however, found only for OBS. Earlier studies have mainly investigated differences only in valence ratings between men and women (Bellezza et al., 1986; Montefinese et al., 2014; Soares et al., 2012; Söderholm et al., 2013; Warriner et al., 2013). Soares et al. (2012) and Warriner et al. (2013), for instance, also found higher valence ratings for males in a Portuguese, respective English version of the ANEW.

It was also shown that the association between VAL and SOC remained significantly positive for both males and females, which is consistent with Britz et al.’s (2019) and Montefinese et al.’s (2014) findings, although overall male participants had higher ratings than female participants. For OBS, a different pattern was found. That is, for males there was a stronger association between both VAL and OBS and SOC and OBS ratings than for females, which may indicate that males perceive a trait as more positively valenced and more socially desirable when it is highly observable. Thus, whereas VAL and SOC did not seem to be affected by the participants’ gender, OBS ratings differed between males and females. Specifically, male participants had higher OBS ratings and a stronger congruence between OBS and both VAL and SOC. Please note, that those gender differences may alternatively be attributed to significant differences in education (see p. 14 - 2.3 *Participants*) although this effect (${\hat{\eta}}_G^2$= 0.005) was of a rather small size.

Previous studies also suggest that gender differences are specific for positive and negative words (Bellezza et al., 1986; Montefinese et al., 2014; Soares et al., 2012; Söderholm et al., 2013), a finding which has been described as valence-specificity (Stevens & Hamann, 2012). A meta-analysis by Stevens and Hamann (2012) found that women were more responsive to negative words and men were more responsive to positive words, as indicated by greater activation of the amygdala. In the current study, this effect was only found for negative words in the SOC and VAL ratings (see Supplements 7). Thus, female participants rated negative words more negatively, respectively as less socially desirable, than the male participants. However, men did not rate the positive words more positively than females (see Table 4).

Further, differences in the age groups were analyzed because previous studies have indicated that older individuals have a stronger preference for positive stimuli than younger individuals (Carstensen et al., 2003), which has been described as the *positivity effect* (Mather & Carstensen, 2005). The socioemotional selectivity theory has offered an explanation for this. It states that an individual’s goals change as a function of their time horizon, whereby older individuals are motivated to focus on the positive aspects in life, whereas younger individuals focus more on learning and exploration (Carstensen & DeLiema, 2018). However, in the current study a significant main effect of age was found only for the OBS ratings. Specifically, the middle-aged participants had the highest OBS ratings, and they were followed by the younger and then the older participants. For the SOC ratings, a negative association was found with age, i.e., the older the participants were, the less socially desirable they rated the traits, although this was not statistically significant. Moreover, the VAL ratings were not significantly related to the participants’ age. There was, nevertheless, a trend toward an increasing consistency in the ratings with increasing age, both for the VAL and the SOC ratings. Previous studies also have reported mixed findings with respect to the association between valence ratings and age (Gilet et al., 2012; Grühn & Smith, 2008; Söderholm et al., 2013). Whereas Grühn and Smith’s (2008) results are in line with the positivity effect, Gilet et al. (2012) reported only small differences in the valence ratings between age groups for French words, with more positive valence ratings given by middle-aged participants compared to younger and older participants. Lastly, Söderholm et al. (2013) found partial support for the positivity effect in valence ratings, which were more positive for positive nouns and more negative for negative nouns in the older participants. Carstensen and DeLiema (2018) reported that the positivity effect has often not been replicated in studies in which participants operate on stimuli (e.g., by giving a rating). The inconsistencies across studies might thus indicate that the positivity effect does not seem to influence the processing of word material. It might, however, also be argued that the failure to replicate the positivity effect in the current study was due to the comparably young age of the sample (Kyröläinen et al., 2021).

### Associations among VAL, SOC, and OBS ratings

In line with previous findings (Britz et al., 2019), a strong association was found between the VAL and SOC ratings. Examination of the top ten words with the highest and lowest VAL and SOC ratings suggests that the constructs show especially high overlap at the scale endpoints (see Table 3). For instance, the adjective *trustworthy* was rated as positive on valence and high on social desirability, whereas the adjectives *traitorous* and *racist* were rated negative on valence and low in social desirability. Further support of the validity of the ratings can be seen in the strong association between the ratings and the psycholinguistic variables. The ratings showed the typical pattern of a positive association with word frequency and a negative association with word length (e.g., Britz et al., 2019; Gilet et al., 2012; Grühn, 2016; Montefinese et al., 2014). Although the results of both the current study and previous findings indicate that social desirability and valence are strongly related, these constructs should still be considered separate in terms of rating content because significant differences between the SOC and VAL absolute mean ratings were found for some words (e.g., *calm*, *childish*, *detached*) that can be found in Database 2 *(Rating Effects*). In addition, VAL and SOC differ for some words, even with respect to the positive or negative rating of the words (so-called divergent words), as can be seen in Table 6, which shows the divergent trait adjectives found in the present study.

There does not seem to be a consensus regarding the directionality of influence, i.e., whether a trait’s social desirability has an effect on its perceived valence or vice versa. Although it has been suggested that a trait’s valence is influenced by the perceived social desirability of a word, i.e., social opinions (Meleddu & Guicciardi, 1998), it might also be speculated that valence and social desirability influence each other. The inclusion of both rating dimensions in the current database will allow researchers to control for a trait’s social desirability when traits are chosen on the basis of their valence. Interestingly, OBS was only moderately related to both SOC and VAL. Despite the moderate correlation, the adjectives displayed in Table 3, which were rated with the highest and lowest observability suggest that some adjectives rated high in observability (e.g., *pretty*, *talkative*, *attractive*) are also associated with high social desirability and positive valence. A less clear relationship was found for the adjective *loud,* which was rated as highly observable but socially undesirable and with a negative valence. The words shown in Table 3, which were rated as difficult to observe in others, were, however, consistently rated low on social desirability and negative in valence (e.g., *unfaithful*: OBS mean = 1.98, VAL mean = – 2.34, SOC mean = – 2.22*;* see Database 1 (*Ratings*) for the VAL, SOC, and OBS ratings means of the words shown in Table 3).

The moderate association between observability and valence is in line with the ratings obtained in Quadflieg et al.’s (2014) French database. These researchers also found no significant associations between the traits’ valence and their observability (Quadflieg et al., 2014). Moreover, previous studies have indicated that trait observability and social desirability can be regarded as separate constructs (Gosling et al., 1998), indicating that traits might, for instance, be considered as highly socially desirable whether they can or cannot be easily observed in a person. In John and Robins’ (1993) study examining the association between observability and social desirability and the agreement between self and peer ratings on the Big Five personality traits, the association between social desirability and observability ratings was also found to be inconsistent. Whereas a trait’s social desirability was highly dependent on the trait’s pole (for instance, words with high poles, e.g., extraversion, were rated as socially desirable, and the words with low poles, e.g., introversion, were rated as socially undesirable), the traits’ observability was rated independently of their pole. Instead, extraversion was rated as most observable (with the high pole rated as even more observable than the low pole), whereas the intellect domain was rated as least observable. Similar to the findings obtained in the current study, John and Robins (1993) found agreement only between desirability and observability on a few personality traits (e.g., extraversion). It might be speculated that the moderate correlation between a trait’s observability and its social desirability at least partially explains the moderate association between valence and observability because, as was argued previously, a trait’s valence is influenced by its social desirability (Meleddu & Guicciardi, 1998). It should, however, be noted that the directionality of the influence between valence and social desirability is still unclear. It would be an interesting endeavor for future research to identify the directionality of influence among the valence, social desirability, and observability dimensions.

Finally, to further confirm the validity of the findings in the current study, the generalizability of the ratings was assessed by means of its associations with the ratings in the ANEW (Bradley & Lang, 1999) and EMOTE (Grühn, 2016) databases, which measure similar concepts. The VAL ratings obtained in the current study were found to be correlated somewhat higher with the valence ratings of the EMOTE database than with the ANEW database, although the correlations with the ratings of both databases were significant. It might be speculated that this finding can be attributed to differences in the rating scales. Whereas Grühn (2016) used a seven-point Likert scale similar to the one used in the current study, Bradley and Lang (1999) used a self-assessment manikin (SAM) on which the participants gave their ratings, which ranged from a smiling, happy figure to a frowning, unhappy figure (Bradley & Lang, 1999, p. 2). The SOC ratings were found to be strongly correlated with the desirability ratings in the EMOTE database. It should, however, be noted that the desirability ratings obtained in the EMOTE database refer to how desirable a characteristic is for oneself (Grühn, 2016) and not how socially desirable a characteristic is in general. It is all the more interesting that social desirability and desirability were strongly associated with each other. In sum, the VAL and SOC ratings were highly congruent with ratings obtained in previous studies.

### Limitations and future research

The split-half reliability and comparability of the current data with other databases indicate a strong consistency in the ratings. Nevertheless, some points remain to be elucidated, which could improve insights in this research area: First, because observability has previously not been included in trait databases gathered from English-speaking samples, the consistency in observability ratings across different studies remains to be elucidated. Similarly, it would be of interest to know the extent to which results vary across cross-cultural samples. Second, the predictive validity of the ratings was not assessed in the current study. Grühn (2016) found that words with a negative valence were remembered better in a memory recognition task than were positive and neutral words. Therefore, it would be of interest for future research to validate the ratings obtained in the current study. Third, the trait adjectives were rated on only three dimensions (VAL, SOC, and OBS) in order to avoid fatigue effects. Because previous studies have shown that additional characteristics might also affect the cognitive processing of word stimuli (Grühn, 2016), future studies could expand the present database by including additional rating dimensions, such as arousal, dominance, imageability, or concreteness. Finally, it might be valuable for future studies to assess reaction times in the ratings of word stimuli as an additional indicator of rating consistency.

### Conclusions

This study aimed to enlarge the German ALoT (Britz et al., 2019) to include trait adjectives in English in order for the database to be more valuable for international researchers. The trait adjectives in English were rated by a larger, more representative sample, thereby allowing us to assess the effects of age and gender on the ratings. Furthermore, by using an online platform and several screening and integrity checks (e.g., non-words) we achieved a more balanced sample and more reliable ratings than we would have otherwise. In addition, observability was added as a third rating dimension, thereby allowing us to identify relationships among the OBS, VAL, and SOC ratings and the demographic characteristics of the participants. The ELoT that we assembled provides researchers with 500 trait adjectives, each of which was rated on SOC, VAL, and OBS and various psycholinguistic variables, such as word frequency and word length. Finally, a tool (https://personality-traits.eu) was provided, which can be used to match stimuli on various attributes that might be of interest or need to be controlled in future experimental studies.

## Supplementary Information

ESM 1 (DOCX 12 kb)

ESM 2 (DOCX 12 kb)

ESM 3 (DOCX 13 kb)

ESM 4 (DOCX 12 kb)

ESM 5 (DOCX 15 kb)

ESM 6 (DOCX 12 kb)

ESM 7 (DOCX 15 kb)

## Footnotes

1 Prior to conducting these analyses, it was determined whether age and gender were equally distributed across the four rating conditions using independent samples *t* tests (see Supplements 6).

## References

[CR1] Ampofo, A. A. (2001). "when men speak women listen": Gender socialisation and young adolescents' attitudes to sexual and reproductive issues. *African Journal of Reproductive Health*, 196–212. 12471941

[CR2] Anderson NH (1968). Likableness ratings of 555 personality-trait words. Journal of Personality and Social Psychology.

[CR3] Ashton MC, Lee K, Goldberg LR (2004). A hierarchical analysis of 1,710 English personality-descriptive adjectives. Journal of Personality and Social Psychology.

[CR4] Aust, F., & Barth, M. (2020). Papaja: Create APA manuscripts with R markdown. *Retrieved from* https://github.com/crsh/papaja

[CR5] Baayen, R., Piepenbrock, R., & Van Rijn, H. (1993). *The CELEX lexical database (CD-ROM) Philadelphia*. PA*:* Linguistic Data Consortium, University of Pennsylvania.

[CR6] Bellezza FS, Greenwald AG, Banaji MR (1986). Words high and low in pleasantness as rated by male and female college students. Behavior Research Methods, Instruments, & Computers.

[CR7] Bliss CA, Kloumann IM, Harris KD, Danforth CM, Dodds PS (2012). Twitter reciprocal reply networks exhibit assortativity with respect to happiness. Journal of Computational Science.

[CR8] Bochner S, Van Zyl T (1985). Desirability ratings of 110 personality-trait words. The Journal of Social Psychology.

[CR9] Bradley, M. M., & Lang, P. J. (1999). *Affective norms for English words (ANEW): Instruction manual and affective ratings (technical report C-1)*. Gainesville, FL: The Center for Research in Psychophysiology, University of Florida.

[CR10] Britz S, Gauggel S, Mainz V (2019). The Aachen list of trait words. Journal of Psycholinguistic Research.

[CR11] Carstensen LL, DeLiema M (2018). The positivity effect: A negativity bias in youth fades with age. Current Opinion in Behavioral Sciences.

[CR12] Carstensen LL, Fung HH, Charles ST (2003). Socioemotional selectivity theory and the regulation of emotion in the second half of life. Motivation and Emotion.

[CR13] Chandler J (2018). Likeableness and meaningfulness ratings of 555 (+ 487) person-descriptive words. Journal of Research in Personality.

[CR14] Charles ST, Piazza JR, Mogle JA, Urban EJ, Sliwinski MJ, Almeida DM (2016). Age differences in emotional well-being vary by temporal recall. Journals of Gerontology Series B: Psychological Sciences and Social Sciences.

[CR15] Costa PT, McCrae RR (1992). Normal personality assessment in clinical practice: The NEO personality inventory. Psychological Assessment.

[CR16] Crowne DP, Marlowe D (1960). A new scale of social desirability independent of psychopathology. Journal of Consulting Psychology.

[CR17] Dill KE, Thill KP (2007). Video game characters and the socialization of gender roles: Young people’s perceptions mirror sexist media depictions. Sex Roles.

[CR18] Fairfield B, Ambrosini E, Mammarella N, Montefinese M (2017). Affective norms for Italian words in older adults: Age differences in ratings of valence, arousal and dominance. PLoS One.

[CR19] Forster SD, Drueke B, Britz S, Gauggel S, Mainz V (2019). How females think about themselves and how they assume that significant others think about them: The influence of perspective taking on self-referential processing. PLoS One.

[CR20] Garcia D, Garas A, Schweitzer F (2012). Positive words carry less information than negative words. EPJ Data Science.

[CR21] Gilet A-L, Grühn D, Studer J, Labouvie-Vief G (2012). Valence, arousal, and imagery ratings for 835 French attributes by young, middle-aged, and older adults: The French emotional evaluation list (FEEL). European Review of Applied Psychology.

[CR22] Gorman AM (1961). Recognition memory for nouns as a function of abstractness and frequency. Journal of Experimental Psychology.

[CR23] Gosling SD, John OP, Craik KH, Robins RW (1998). Do people know how they behave? Self-reported act frequencies compared with on-line codings by observers. Journal of Personality and Social Psychology.

[CR24] Grühn D (2016). An English word database of EMOtional TErms (EMOTE). Psychological Reports.

[CR25] Grühn D, Sharifian N (2016). Lists of emotional stimuli. *Emotion measurement*.

[CR26] Grühn D, Smith J (2008). Characteristics for 200 words rated by young and older adults: Age-dependent evaluations of German adjectives (AGE). Behavior Research Methods.

[CR27] Hager, W., & Hasselhorn, M. (1994). Über Variablen, die eingeschätzt werden sollen, und über Variablen, die eingeschätzt werden: Emotionalität, Angenehmheit, Prägnanz, Erwünschtheit und Sympathie [on variables that should be estimated and variables that are estimated: Emotionality, pleasantness, meaningfulness, desirability, and likability]. *Handbuch deutschsprachiger Wortnormen*, 226–248.

[CR28] Holtgraves T (2004). Social desirability and self-reports: Testing models of socially desirable responding. Personality and Social Psychology Bulletin.

[CR29] Human LJ, Biesanz JC (2011). Target adjustment and self-other agreement: Utilizing trait observability to disentangle judgeability and self-knowledge. Journal of Personality and Social Psychology.

[CR30] Jacobson LI, Kellogg RW, Cauce AM, Slavin RS (1977). A multidimensional social desirability inventory. Bulletin of the Psychonomic Society.

[CR31] John OP, Robins RW (1993). Determinants of interjudge agreement on personality traits: The big five domains, observability, evaluativeness, and the unique perspective of the self. Journal of Personality.

[CR32] Kanske P, Kotz SA (2010). Leipzig affective norms for German: A reliability study. Behavior Research Methods.

[CR33] Kauschke C, Bahn D, Vesker M, Schwarzer G (2019). The role of emotional valence for the processing of facial and verbal stimuli—Positivity or negativity bias?. Frontiers in Psychology.

[CR34] Konstabel K, Aavik T, Allik J (2006). Social desirability and consensual validity of personality traits. European Journal of Personality.

[CR35] Kyröläinen, A. J., Luke, J., Libben, G., & Kuperman, V. (2021). Valence norms for 3,600 English words collected during the COVID-19 pandemic: Effects of age and the pandemic. *Behavior Research Methods*, 1–12. 10.3758/s13428-021-01740-0 PMC8676940 34918233

[CR36] Lahl O, Göritz AS, Pietrowsky R, Rosenberg J (2009). Using the world-wide web to obtain large-scale word norms: 190,212 ratings on a set of 2,654 German nouns. Behavior Research Methods.

[CR37] Lalwani AK, Shrum L, Chiu C-Y (2009). Motivated response styles: The role of cultural values, regulatory focus, and self-consciousness in socially desirable responding. Journal of Personality and Social Psychology.

[CR38] Mainz V, Britz S, Forster SD, Drüke B, Gauggel S (2020). Transcranial direct current stimulation of the medial prefrontal cortex has no specific effect on self-referential processes. Frontiers in Human Neuroscience.

[CR39] Mather M, Carstensen LL (2005). Aging and motivated cognition: The positivity effect in attention and memory. Trends in Cognitive Sciences.

[CR40] Meleddu M, Guicciardi M (1998). Self-knowledge and social desirability of personality traits. European Journal of Personality.

[CR41] Mohammad, S. (2018). *Obtaining reliable human ratings of valence, arousal, and dominance for 20,000 English words.* Paper presented at the Proceedings of the 56th Annual Meeting of the Association for Computational Linguistics (Volume 1: Long Papers).

[CR42] Montefinese M, Ambrosini E, Fairfield B, Mammarella N (2014). The adaptation of the affective norms for English words (ANEW) for Italian. Behavior Research Methods.

[CR43] Moran JM, Lee SM, Gabrieli JD (2011). Dissociable neural systems supporting knowledge about human character and appearance in ourselves and others. Journal of Cognitive Neuroscience.

[CR44] Olejnik S, Algina J (2003). Generalized eta and omega squared statistics: Measures of effect size for some common research designs. Psychological Methods.

[CR45] Quadflieg S, Michel C, Bukowski H, Samson D (2014). A database of psycholinguistic and lexical properties for French adjectives referring to human and/or nonhuman attributes. Canadian Journal of Experimental Psychology/Revue canadienne de psychologie expérimentale.

[CR46] R Core Team. (2021). *R: A language and environment for statistical computing*. Vienna, Austria: R Foundation for Statistical Computing Retrieved from https://www.R-project.org/

[CR47] Ric F, Alexopoulus T, Müller D, Aubé B (2013). Emotional norms for 524 French personality trait words. Cognition and Emotion.

[CR48] Roberts C, Freeman J, Samdal O, Schnohr CW, De Looze M, Gabhainn SN, Rasmussen M (2009). The health behaviour in school-aged children (HBSC) study: Methodological developments and current tensions. International Journal of Public Health.

[CR49] Rosen E (1956). Self-appraisal, personal desirability, and perceived social desirability of personality traits. The Journal of Abnormal and Social Psychology.

[CR50] Rubin DC, Friendly M (1986). Predicting which words get recalled: Measures of free recall, availability, goodness, emotionality, and pronunciability for 925 nouns. Memory & Cognition.

[CR51] Schmidtke DS, Schröder T, Jacobs AM, Conrad M (2014). ANGST: Affective norms for German sentiment terms, derived from the affective norms for English words. Behavior Research Methods.

[CR52] Soares AP, Comesaña M, Pinheiro AP, Simões A, Frade CS (2012). The adaptation of the affective norms for English words (ANEW) for European Portuguese. Behavior Research Methods.

[CR53] Söderholm C, Häyry E, Laine M, Karrasch M (2013). Valence and arousal ratings for 420 Finnish nouns by age and gender. PLoS One.

[CR54] Stevens JS, Hamann S (2012). Sex differences in brain activation to emotional stimuli: A meta-analysis of neuroimaging studies. Neuropsychologia.

[CR55] Stöber J (2001). The social desirability Scale-17 (SDS-17): Convergent validity, discriminant validity, and relationship with age. European Journal of Psychological Assessment.

[CR56] Team, R. C. R: A language and environment for statistical computing. 2021; Viewed 20 April 2021. *Reference Source*.

[CR57] Van Heuven WJ, Mandera P, Keuleers E, Brysbaert M (2014). SUBTLEX-UK: A new and improved word frequency database for British English. Quarterly Journal of Experimental Psychology.

[CR58] Vazire S (2010). Who knows what about a person? The self–other knowledge asymmetry (SOKA) model. Journal of Personality and Social Psychology.

[CR59] Verheyen S, De Deyne S, Linsen S, Storms G (2020). Lexicosemantic, affective, and distributional norms for 1,000 Dutch adjectives. Behavior Research Methods.

[CR60] Võ ML, Jacobs AM, Conrad M (2006). Cross-validating the Berlin affective word list. Behavior Research Methods.

[CR61] Warriner AB, Kuperman V, Brysbaert M (2013). Norms of valence, arousal, and dominance for 13,915 English lemmas. Behavior Research Methods.

[CR62] Watson D, Hubbard B, Wiese D (2000). Self–other agreement in personality and affectivity: The role of acquaintanceship, trait visibility, and assumed similarity. Journal of Personality and Social Psychology.

[CR63] Wentura D, Rothermund K, Bak P (2000). Automatic vigilance: The attention-grabbing power of approach-and avoidance-related social information. Journal of Personality and Social Psychology.

[CR64] Whissell C (1989). The dictionary of affect in language. *The measurement of emotions*.

[CR65] Whissell C (2009). Using the revised dictionary of affect in language to quantify the emotional undertones of samples of natural language. Psychological Reports.

[CR66] Wood D, Nye CD, Saucier G (2010). Identification and measurement of a more comprehensive set of person-descriptive trait markers from the English lexicon. Journal of Research in Personality.

[CR67] Xie Y (2019). TinyTeX: A lightweight, cross-platform, and easy-to-maintain LaTeX distribution based on TeX live. TUGboat.

