# Supplementary material for: An English list of trait words including valence, social desirability, and observability ratings
Source: Behav Res Methods. 2022 Aug 12;55(5):2669–86. doi: 10.3758/s13428-022-01921-5 (PMC10439032; doi:10.3758/s13428-022-01921-5)
Supplement: Supplementary file 1 — (DOCX 12 kb) [file 13428_2022_1921_MOESM1_ESM.docx]

**Supplements 1 – Comparison of word lengths and frequency between word list 1 and 2**

**Data analysis**

The 500 trait adjectives were randomly split into two word lists based on letter lengths and alphabetical distribution. Since previous studies showed that psychometric characteristics such as word lengths and frequency can affect valence and social desirability ratings (e.g., Britz et al., 2019), independent samples t-Tests were conducted to assess whether word lengths and frequency were equally distributed across word list 1 and 2.

**Results**

No significant differences were found between word list 1 and 2 regarding word length (t(498) = .199, *p* = .842), and frequency (Celex log_10_: *t*(366) = -.910, *p* = .363; SUBTLEX Zipf: *t*(470) = -.323, *p* = .746).
