# Supplementary material for: An English list of trait words including valence, social desirability, and observability ratings
Source: Behav Res Methods. 2022 Aug 12;55(5):2669–86. doi: 10.3758/s13428-022-01921-5 (PMC10439032; doi:10.3758/s13428-022-01921-5)
Supplement: Supplementary file 2 — (DOCX 12 kb) [file 13428_2022_1921_MOESM2_ESM.docx]

**Supplements 2 – List of non-words**

Table: *List of non-words and number of participants indicating the word as non-word*

| **Non-word** | **N (%)** |
| --- | --- |
| northeloomed | 758 (92.21%) |
| redriff | 734 (89.29%) |
| waystaily | 721 (87.71%) |
| aucrazed | 736 (89.53%) |
| fairise | 707 (86.00%) |
| exorph | 748 (90.99%) |
| puncert | 738 (89.78%) |
| efully | 755 (91.84%) |
| eakfoo | 785 (95.49%) |
| peccin | 771 (93.79%) |

*Note*. N (%)=Number (percentage) of participants who indicated “I don’t know the meaning of the word” for this non-word
