# Supplementary material for: An English list of trait words including valence, social desirability, and observability ratings
Source: Behav Res Methods. 2022 Aug 12;55(5):2669–86. doi: 10.3758/s13428-022-01921-5 (PMC10439032; doi:10.3758/s13428-022-01921-5)
Supplement: Supplementary file 3 — (DOCX 13 kb) [file 13428_2022_1921_MOESM3_ESM.docx]

**Supplements 3 – Sequence effects**

**Data analysis**

Before the actual analyses were performed, it was examined whether the presentation order of the conditions had an effect on the VAL, SOC and OBS ratings. To this end, independent sample *t*-Tests were conducted per sample and condition comparing the two presentation orders. More specifically, in the VAL-OBS conditions, the presentation orders VAL-OBS vs. OBS-VAL were compared for VAL and OBS ratings whereas in the SOC-OBS conditions the presentation orders SOC-OBS vs. OBS-SOC were compared for SOC and OBS ratings for word list 1 and 2 resulting in a total of 8 independent samples *t*-Tests. In addition, correlations were calculated using Pearson`s r per condition to examine the association between the ratings per presentation order.

**Results**

The independent sample *t*-Tests revealed that the ratings were not significantly influenced by the sequence the participants were in. Regarding word list 1, no significant differences were found between the two presentation orders (VAL: (*t*(498) = -.16, *p* = .876) & OBS (*t*(498) = 1.94, *p* = .053); SOC: (*t*(498) = .15, *p* = .881) & OBS: (*t*(498) = 1.35, *p* = .177)). Similar results were found for word list 2 (VAL: (*t*(498) = -.01, *p* = .993) & OBS (*t*(498) = 1.56, *p* = .120); SOC: (*t*(498) = .24, *p* = .809) & OBS: (*t*(498) = .92, *p* = .356)).

Furthermore, high correlations were found within each condition of wordlist 1 (VAL (*r* = .99, 95% CI [.99, .99]) & OBS (*r* = .94, 95% CI [.92, .95])); SOC (*r* > .99, 95% CI [.99, >.99]) & OBS (*r* = .95, 95% CI [.93, .96]), and wordlist 2 (VAL (*r* > .99, 95% CI [.99, >.99]) & OBS (*r* = .94, 95% CI [.92, .95])); SOC (*r* = .99, 95% CI [.99, >.99]) & OBS (*r* = .94, 95% CI [.93, .95])).

The following analyses of the ratings were therefore conducted outgoing from an independence of the presentation order.
