# Supplementary material for: An English list of trait words including valence, social desirability, and observability ratings
Source: Behav Res Methods. 2022 Aug 12;55(5):2669–86. doi: 10.3758/s13428-022-01921-5 (PMC10439032; doi:10.3758/s13428-022-01921-5)
Supplement: Supplementary file 4 — (DOCX 12 kb) [file 13428_2022_1921_MOESM4_ESM.docx]

**Supplements 4 – Breakdown of dropouts**

The breakdown of the total number of screened out participants based on the Qualtrics sampling procedure, the comprehension checks and the post-hoc data integrity checks is as follows:

In total, the survey was accessed 15738 times by different platform attendees.

Based on the prerequisites (nationality, age, no mobile phone participation) 11547 participants were screened out (screening criteria: 1297, quotas: 2576, quality checks: 7674).

A total of 2990 participants started but terminated the study voluntarily at some point.

Based on the post-hoc data integrity checks further 379 participants were screened out.

No participant had to be excluded due to a too long response time (> 12 h).

A final sample of 822 participants completed the study.
