# Supplementary material for: An English list of trait words including valence, social desirability, and observability ratings
Source: Behav Res Methods. 2022 Aug 12;55(5):2669–86. doi: 10.3758/s13428-022-01921-5 (PMC10439032; doi:10.3758/s13428-022-01921-5)
Supplement: Supplementary file 5 — (DOCX 15 kb) [file 13428_2022_1921_MOESM5_ESM.docx]

**Supplements 5 – Top 10 trait adjectives**

Table: *Top 10 trait adjectives for VAL, SOC and OBS of the total sample, females and males*

|  | **VAL** | | | **SOC** | | | **OBS** | | |
| --- | --- | --- | --- | --- | --- | --- | --- | --- | --- |
| Sample | Words | Mean | SD | Words | Mean | SD | Words | Mean | SD |
| Total (N = 822) | loving | 2.58 | 0.76 | trustworthy | 2.69 | 0.73 | loud | 3.76 | 0.59 |
|  | honest | 2.58 | 0.67 | hardworking | 2.56 | 0.89 | pretty | 3.71 | 0.57 |
|  | trustworthy | 2.35 | 0.82 | helpful | 2.55 | 0.72 | attractive | 3.7 | 0.53 |
|  | compassionate | 2.45 | 0.76 | respectable | 2.51 | 0.71 | talkative | 3.7 | 0.56 |
|  | reliable | 2.43 | 0.76 | friendly | 2.5 | 0.82 | beautiful | 3.67 | 0.59 |
|  | traitorous | -2.62 | 0.77 | tyrannical | -2.52 | 1.07 | lonely | 2.16 | 1 |
|  | hateful | -2.64 | 0.64 | traitorous | -2.53 | 1.1 | traitorous | 2.14 | 1.04 |
|  | corrupt | -2.67 | 0.68 | malicious | -2.55 | 0.88 | sly | 2.14 | 0.89 |
|  | cruel | -2.67 | 0.67 | violent | -2.66 | 0.88 | underhand | 2.11 | 0.92 |
|  | racist | -2.8 | 0.58 | racist | -2.7 | 0.94 | unfaithful | 1.98 | 1.03 |
| Female (N = 472) | honest | 2.64 | 0.63 | trustworthy | 2.66 | 0.81 | studious | 3.74 | 0.48 |
|  | loving | 2.64 | 0.75 | honest | 2.6 | 0.67 | talkative | 3.74 | 0.48 |
|  | compassionate | 2.53 | 0.73 | dependable | 2.58 | 0.81 | prudish | 3.7 | 0.58 |
|  | trustworthy | 2.53 | 0.83 | respectful | 2.58 | 0.81 | pretty | 3.7 | 0.58 |
|  | reliable | 2.49 | 0.69 | kind | 2.57 | 0.81 | astute | 3.57 | 0.54 |
|  | hateful | -2.65 | 0.59 | cruel | -2.62 | 0.87 | lonely | 1.99 | 0.92 |
|  | cruel | -2.69 | 0.71 | corrupt | -2.64 | 0.88 | timid | 1.98 | 0.85 |
|  | corrupt | -2.7 | 0.62 | traitorous | -2.65 | 0.78 | underhand | 1.98 | 0.85 |
|  | violent | -2.77 | 0.54 | racist | -2.77 | 0.8 | tough | 1.82 | 0.94 |
|  | racist | -2.77 | 0.68 | violent | -2.78 | 0.72 | unfaithful | 1.82 | 0.94 |
| Male (N = 350) | loving | 2.51 | 0.76 | trustworthy | 2.74 | 0.62 | prudish | 3.72 | 0.56 |
|  | honest | 2.49 | 0.72 | hardworking | 2.57 | 0.8 | pretty | 3.72 | 0.56 |
|  | respectful | 2.48 | 0.61 | helpful | 2.55 | 0.66 | studious | 3.64 | 0.64 |
|  | trustworthy | 2.44 | 0.81 | respectable | 2.52 | 0.7 | talkative | 3.64 | 0.64 |
|  | happy | 2.42 | 0.87 | educated | 2.5 | 0.68 | philosophical | 3.57 | 0.61 |
|  | hateful | -2.62 | 0.71 | deceitful | -2.48 | 0.83 | traitorous | 2.24 | 1.12 |
|  | corrupt | -2.62 | 0.76 | malicious | -2.48 | 0.91 | tough | 2.19 | 1.1 |
|  | cruel | -2.64 | 0.61 | violent | -2.49 | 1.04 | unfaithful | 2.19 | 1.1 |
|  | traitorous | -2.7 | 0.62 | tyrannical | -2.56 | 1.05 | merciful | 2.18 | 1 |
|  | racist | -2.85 | 0.42 | racist | -2.61 | 1.09 | mercenary | 2.18 | 1 |
