# Supplementary material for: An English list of trait words including valence, social desirability, and observability ratings
Source: Behav Res Methods. 2022 Aug 12;55(5):2669–86. doi: 10.3758/s13428-022-01921-5 (PMC10439032; doi:10.3758/s13428-022-01921-5)
Supplement: Supplementary file 6 — (DOCX 12 kb) [file 13428_2022_1921_MOESM6_ESM.docx]

**Supplements 6 – Distribution of demographic variables across rating conditions**

**Data analysis**

To be able to compare the effects of age and gender in the VAL, SOC and OBS ratings, it was first investigated whether age and gender were approximately equally distributed across participants who rated word list 1 and 2. To assess significant differences with regard to age, independent sample *t*-Tests were conducted for each rating (VAL, SOC and OBS) between the participants who rated word lists 1 and 2. To assess whether the participants of word list 1 and 2 significantly differed with regards to gender, Chi-square tests were conducted for each rating.

**Results**

Both age (*t*(413) = .70, *p* = .487) and gender ($\chi^{2}$(1,407) = .02, *p* = .886) did not significantly differ between word lists 1 and 2 for those who rated VAL and OBS. Similarly, for those who rated SOC and OBS, again, no significant differences were found between word lists 1 and 2 for age (*t*(405) = -.60, *p* = .552) and gender ($\chi^{2}$(1,415) = .00, *p* > .999).
