# Supplementary material for: An English list of trait words including valence, social desirability, and observability ratings
Source: Behav Res Methods. 2022 Aug 12;55(5):2669–86. doi: 10.3758/s13428-022-01921-5 (PMC10439032; doi:10.3758/s13428-022-01921-5)
Supplement: Supplementary file 7 — (DOCX 15 kb) [file 13428_2022_1921_MOESM7_ESM.docx]

**Supplements 7 – Gender and age group differences in response distributions**

**Data analyses**

To assess gender differences in the rating conditions’ rating scale usage, first, the rating values > 0 for VAL and SOC, and > 2.5 for OBS were defined as “high” and negative ratings values < 0 for VAL and SOC and < 2.5 for OBS were defined as “low” rating expression.

Three 2x2 ANOVAs were run with gender (male versus female) as between subject factor, the rating expression (high versus low) as within-factor and the rating values (VAL, SOC or OBS) across all 500 words were entered as the dependent variables. The values used for these analyses refer to Database 3 (*Gender effects*, e.g., VAL FEMALE MEAN, VALE MALE MEAN; please note, that the values of database 3 were then further split into “high” and “low” rating expression).

Likewise, to assess whether the three age groups differed with regard to the rating scale usage three 3x2 ANOVAs were conducted for each rating (VAL, SOC and OBS) with age group (*younger*, *middle-aged, older*) as between subject factor, rating expression (high versus low) as within-factor and the rating values (VAL, SOC or OBS) across all 500 words were entered as the dependent variables, respectively. The values used for these analyses refer to Database 4 (*Age effects*, e.g. VAL AGE 18-34 MEAN, VAL AGE 35-49 MEAN, VAL AGE 50-65 MEAN; please note, that the values of database 4 were then further split into “high” and “low” rating expression). Post-hoc comparisons were conducted using Tukey tests.

For all analyses, the mean square of error (*MSE*) will be reported as well as generalized eta squared effect sizes ($\hat{\eta}_{G}^{2}$) and the respective confidence intervals.

**Results**

The ANOVA assessing gender differences in the rating scale usage in the VAL rating revealed a non-significant main effect for gender (*F*(1,998) = 0.02, *MSE* = 0.43, *p* = .882, $\hat{\eta}_{G}^{2}$=.000) and a significant main effect of rating expression (*F*(1,998) = 4,577.62, *MSE* = 0.43, *p* < .001, $\hat{\eta}_{G}^{2}$ = .821). The interaction was non-significant (*F*(1,998) = 3.30, *MSE* = 0.43, *p* = .070, $\hat{\eta}_{G}^{2}$ = .003). The ANOVA assessing gender differences in the rating scale usage in the SOC rating showed a non-significant main effect for gender (*F*(1,998) = 0.22, *MSE* = 0.44, *p* = .636, $\hat{\eta}_{G}^{2}$ = .000) and a significant main effect for rating expression (*F*(1,998) = 5,367.81, *MSE* = 0.44, *p* < .001, $\hat{\eta}_{G}^{2}$ = .843). The interaction was also significant (*F*(1,998) = 8.57, *MSE* = 0.44, *p* = .003, $\hat{\eta}_{G}^{2}$ = .009). For the OBS ratings, a non-significant main effect of gender (*F*(1,998) = 1.98, *MSE* = 0.07, *p* = .160, $\hat{\eta}_{G}^{2}$ = .002) and a significant main effect for rating expression (*F*(1,998) = 564.69, *MSE* = 0.07, *p* < .001, $\hat{\eta}_{G}^{2}$ = .361) were found in the ANOVA assessing gender differences in the rating scale usage in the OBS ratings. The interaction was not significant (*F*(1,998) = 0.41, *MSE* = 0.07, *p* = .522, $\hat{\eta}_{G}^{2}$ = .000).

The ANOVA assessing age group differences in the rating scale usage in the VAL rating revealed a non-significant main effect for age group (*F*(1,1,499) = 0.39, *MSE* = 0.43, *p* = .532, $\hat{\eta}_{G}^{2}$ = .000) and a significant main effect of rating expression (*F*(1,1,499) = 6,827.28, *MSE* = 0.43, *p* < .001, $\hat{\eta}_{G}^{2}$ = .820). The interaction was non-significant (*F*(1,1,499) = 2.90, *MSE* = 0.43, *p* = .089, $\hat{\eta}_{G}^{2}$ = .002). Similarly, the ANOVA assessing gender differences in the rating scale usage in the SOC rating also showed a non-significant main effect for age group (*F*(1,1,499) = 1.92, *MSE* = 0.45, *p* = .166, $\hat{\eta}_{G}^{2}$ = .001) and a significant main effect of rating expression (*F*(1,1,499) = 7,895.99, *MSE* = 0.45, *p* < .001 $\hat{\eta}_{G}^{2}$ = .840). The interaction was, however, found to be significant (*F*(1,1,499) = 11.24, *MSE* = 0.45, *p* = .001, $\hat{\eta}_{G}^{2}$ = .007). Lastly, a non-significant main effect of age group (*F*(1,1,499) = 2.61, *MSE* = 0.07, *p* = .107, $\hat{\eta}_{G}^{2}$ = .002) and a significant main effect for rating expression (*F*(1,1,499) = 848.69, *MSE* = 0.07, *p* < .001, $\hat{\eta}_{G}^{2}$ = .361) were found in the ANOVA assessing age group differences in the rating scale usage in the OBS ratings. The interaction was not significant (*F*(1,1,499) = 0.12, *MSE* = 0.07, *p* = .727, $\hat{\eta}_{G}^{2}$ = .000).
